# Change in ocean subsurface environment to suppress tropical cyclone intensification under global warming

**DOI:** 10.1038/ncomms8188

**Published:** 2015-05-18

**Authors:** Ping Huang, I. -I Lin, Chia Chou, Rong-Hui Huang

**Affiliations:** 1 Center for Monsoon System Research, Institute of Atmospheric Physics, Chinese Academy of Sciences, Beijing 100190, China; 2 Joint Center for Global Change Studies (JCGCS), Beijing 100875, China; 3 Department of Atmospheric Sciences, National Taiwan University, Taipei 10617, Taiwan; 4 Research Center for Environmental Changes, Academia Sinica, Taipei 11529, Taiwan

## Abstract

Tropical cyclones (TCs) are hazardous natural disasters. Because TC intensification is significantly controlled by atmosphere and ocean environments, changes in these environments may cause changes in TC intensity. Changes in surface and subsurface ocean conditions can both influence a TC's intensification. Regarding global warming, minimal exploration of the subsurface ocean has been undertaken. Here we investigate future subsurface ocean environment changes projected by 22 state-of-the-art climate models and suggest a suppressive effect of subsurface oceans on the intensification of future TCs. Under global warming, the subsurface vertical temperature profile can be sharpened in important TC regions, which may contribute to a stronger ocean coupling (cooling) effect during the intensification of future TCs. Regarding a TC, future subsurface ocean environments may be more suppressive than the existing subsurface ocean environments. This suppressive effect is not spatially uniform and may be weak in certain local areas.

## Abstract (web summary)

Changing atmospheric and oceanic environments under the effects of a warming climate are likely to influence the intensity of tropical cyclones. Here, the authors analyse 22 state-of-the-art climate models and show that future subsurface ocean environments might further suppress cyclone intensification.

## Graphical abstract

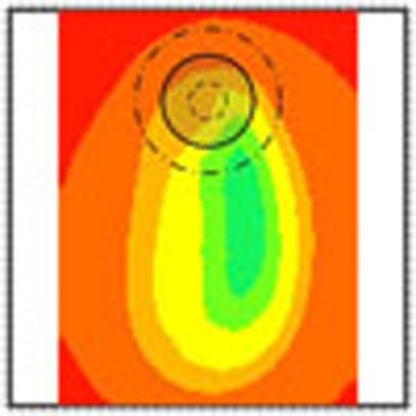

Tropical cyclones (TCs) are significant natural disasters that impose a threat to a global population of almost a billion people^1^. A heightened understanding of TC activities (for example, frequency, track, rainfall, intensification, landfall and surge), especially in the context of global warming, is necessary^2^,^3^,^4^,^5^,^6^,^7^,^8^,^9^,^10^,^11^,^12^,^13^,^14^,^15^,^16^,^17^,^18^,^19^. Because TCs are significantly controlled by their surrounding atmosphere and ocean environments^20^,^21^,^22^,^23^,^24^,^25^,^26^,^27^,^28^,^29^,^30^, changes in future environmental conditions may contribute to possible changes in TC activities. In this study, we investigate the changes in ocean environments (including subsurface ocean changes) under global warming^9^,^13^,^16^,^17^ and the possible impact on future TCs.

The ocean is the source of energy supply for a TC's intensification. The surface and the upper subsurface ocean (typically, from the surface to a depth in the range of 100–200 m) are important^22^,^23^,^24^,^25^,^26^,^31^. However, few studies have addressed the subsurface ocean aspect in global warming research^9^,^13^. Energy is supplied from the ocean surface to a TC via air–sea sensible and latent heat fluxes for intensification. The subsurface ocean is usually colder than the surface ocean^22^,^23^,^24^,^25^,^26^,^31^. During a TC's intensification, the intense TC wind inevitably mixes the pre-existing and colder subsurface water with the ocean surface to reduce the sea surface temperature (SST), which is referred to as the TC-induced ocean cooling (coupling) effect (referred to as OCE)^22^,^23^,^24^,^25^,^26^,^31^. OCE is a function of the initial ocean condition (that is, vertical temperature profile), TC intensity, TC travelling speed and TC size^22^,^23^,^24^,^25^,^26^,^27^,^28^,^31^.

The stronger the OCE during a TC's intensification, the colder the during-TC SST and the smaller are the available air–sea sensible and latent heat fluxes for TC intensification^21^,^22^,^23^,^28^,^29^,^30^. Thus, the OCE is a well-known suppressor for constraining a TC's intensification^21^,^22^,^23^,^24^,^25^,^26^,^27^,^28^,^29^,^30^,^31^. However, in the context of global warming, it is still unclear to what extent the ocean surface and subsurface environment will change in important TC regions and whether the consequential changes in the OCE are significant to impact future TCs.

To address these issues, a TC–ocean ‘coupled' approach is needed; the commonly employed ‘uncoupled' approach^6^,^9^ is inadequate as a subsurface ocean is artificially excluded in the uncoupled approach to reduce complexity. Although the SST with OCE is the actual SST encountered by a TC during its intensification (a dynamical process), the uncoupled approach does not exhibit an OCE and the SST is artificially fixed (that is, no reduction) at the pre-TC level.

Few coupled TC projections are included in existing global warming literature. In a pioneering study by Knutson *et al*.^2^, ocean data from the Coupled Model Intercomparison Project Phase 2 (CMIP2) were employed as the initial condition. A minor impact from changes in the subsurface ocean on TC intensity change was suggested. Knutson *et al*.^2^ recommended a reinvestigation when newer-generation ocean data with improved ocean simulations are available. In recent years, Emanuel^12^ conducted coupled projections; however, because the ocean subsurface initial condition is fixed at the present time (that is, no change under global warming), the associated impact cannot be assessed. The dynamical-downscaling approaches of Bender *et al*.^8^ and Knutson *et al*.^13^ are based on the earlier-generation CMIP3 multi-model ensemble (MME) ocean field (that is, without assessing the spread among individual models of ocean vertical stratification changes) and are coupled to the CMIP3 or CMIP5 atmospheric fields. Therefore, a new assessment that incorporates both the ocean fields and the atmospheric fields from recent CMIP5 data^32^ and includes the ensemble member spread to obtain uncertainty estimation is desirable.

In this study, we use the projected atmosphere and ocean fields from the 22 CMIP5 climate models (see Methods and Supplementary Table 1)^32^. Ocean environments in current and global warming conditions are compared. The study regions comprise two important TC basins—the western North Pacific (WNP) and the North Atlantic (NA)^1^. In these TC-active oceans, the pre-TC–ocean environment is found to change considerably under global warming. Although both surface oceans and subsurface oceans warm, subsurface oceans warm at a slower rate than surface oceans over large regions. This differential warming can sharpen future subsurface temperature gradient. This signal is robust across the ensemble members. At representative stations, 17 out of 22 (77%) members over the WNP and 21 out of 22 members (95%) over the NA, exhibit sharpening. This sharpening in ocean's pre-existing vertical temperature gradient can contribute to a stronger OCE (estimated using the same TC attributes) under global warming. The change in subsurface vertical temperature gradient and its interaction with TC suggests that possible negative feedback (damper) from subsurface ocean may exist to constrain TC intensification under global warming.

## Results

### Changes in ocean environments

**Figure 1:**
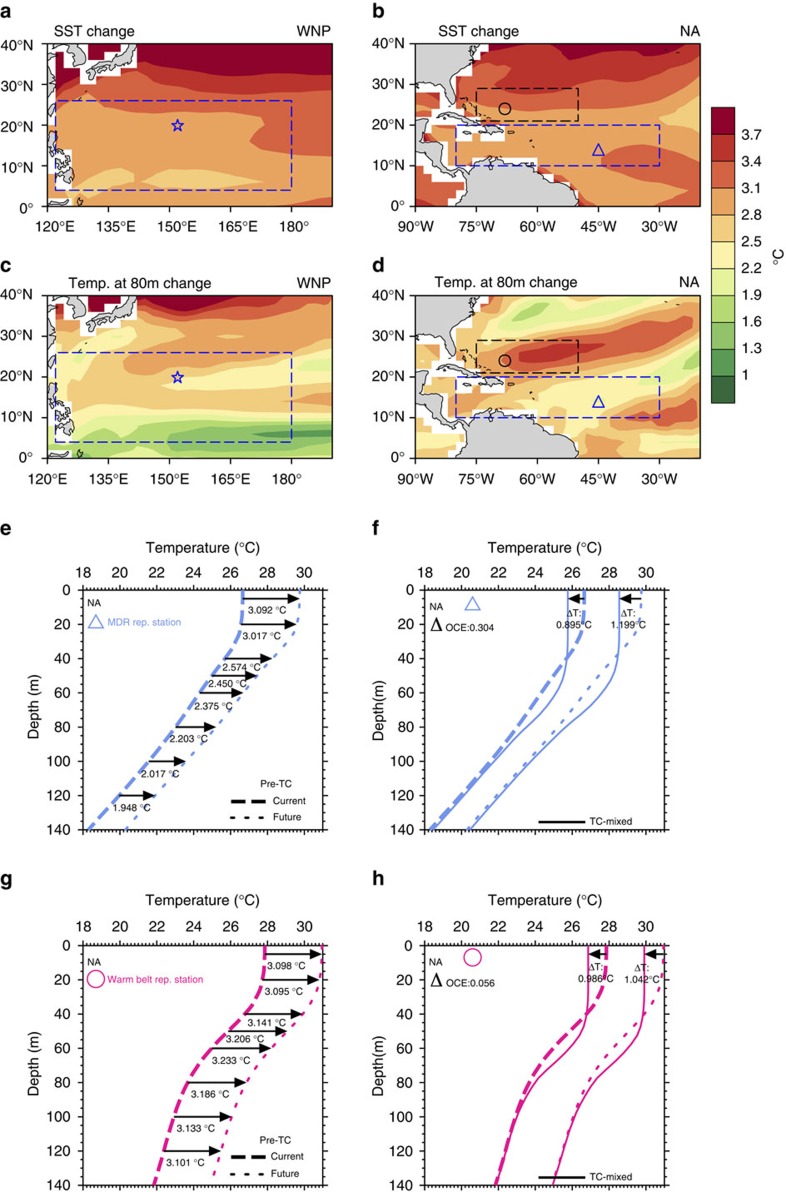
Ocean environment change and influences on the TC-induced OCE. (**a**,**b**) SST warming over the WNP and the NA. (**c**,**d**) Similar to **a**,**b**, but for the ocean subsurface temperature at 80 m. The change is defined as the difference between the 2091–2100 mean and the 2006–2015 mean. The two large boxes denote the MDRs over the WNP and the NA. The NA warm belt region is denoted by a smaller box (northern box) in **b**,**d**. The star, triangle and circle denote the positions of the selected representative stations for the three regions. (**e**,**f**) Initial (**e**) and TC-mixed (**f**, output from the 3DPWP for scenario 8) ocean temperature profiles for the representative station of the NA MDR, comparing current and future conditions. (**g**,**h**) Similar to **e**,**f**, but for the NA warm belt. In **e**,**g**, the arrows and numbers denote the initial temperature increases under global warming at various ocean depths. Rep,. representative; Temp., temperature.

More than a decade ago, Knutson *et al*.^2^ examined changes in the initial ocean environment due to global warming using the CMIP2 ocean field. They reported sharpening in the ocean thermal gradient (increased stratification) due to global warming compared with the existing environment. This finding may be explained by the global warming condition, the increasing net downward heat flux driven by increasing CO_2_, which initially heats the surface waters and subsequently heats the subsurface waters^2^,^16^. Ocean subsurface waters warm at a slower rate than surface waters. As a result, the ocean depth–temperature gradient sharpens. This general stratification increase is also discussed in global studies by oceanographers, in which CMIP3 data are analysed^17^. This change in ocean stratification can significantly impact air–sea biogeochemical processes as well^17^. In this study, we examined the situation using recent CMIP5 data (Representative Concentration Pathway 8.5)^32^. Figure 1 depicts the MME results. The results from the 22 individual model members are detailed in Supplementary Figs 1–3. As shown in Fig. 1a,b, future (2092–2100 average) SSTs in the WNP and NA increase by ∼2.8–4.0 °C with respect to existing SSTs (2006–2014). However, the subsurface ocean warms at a significantly lower rate. For example, the range of warming at a depth of 80 m was ∼1.9–3.4 °C, that is, 15–30% less (Fig. 1c,d).

This differential warming between the surface ocean and the subsurface ocean considerably sharpens the future ocean vertical temperature gradient and stratification increases (Fig. 1e and Supplementary Figs 1, 2 and 4). This sharpening is observed not only in the MME but also in the majority of the individual ensemble members. In the representative station of the WNP TC main development region (MDR, Fig. 1c), 17 of the 22 (77%) members exhibit this sharpening (Supplementary Fig. 1c); in the NA MDR representative station (Fig. 1d), 21 of the 22 members (95%, Supplementary Fig. 2c). Compared with the earlier-generation CMIP2 profiles in Knutson *et al*.^2^, the gradient sharpening from the latest CMIP5 profiles is much more evident than the CMIP2 profiles (Supplementary Figs 5 and 6).

In addition to the general sharpening in the ocean stratification, some localized variability exists. For example, at ∼20–30° N of the NA (northern box in Fig. 1d), the subsurface warming is similar to the SST. Throughout this region, the subsurface warms as much as the SST and minimal stratification sharpening is observed (referred to as the warm belt region, Fig. 1b,d,g).

### Consequential changes in OCE

The previous results suggest that future ocean environments may substantially differ from existing ocean environments in the WNP and NA. Given this change in the initial ocean environment due to global warming, we conduct a series of numerical experiments to assess the consequential impact on the OCE.

Figure 1f provides a simple illustration to explain the concept of OCE^31^,^33^. The two left profiles consist of the current profile pair—the first profile represents the initial pre-TC condition (dashed profile) and the second profile represents the during-TC TC-mixed condition (solid profile). The OCE in the existing condition is 0.895 °C because it is the reduction in SST from the pre-TC SST to the TC-mixed SST. Similarly, the two right profiles consist of the future profile pair and the corresponding OCE is 1.199 °C. In this example, the existing OCE is 0.895 °C, and the future OCE is 1.199 °C. Thus, the OCE increases by 0.304 °C in the future, based on this example.

As previously discussed, the OCE is a function of both ocean and TC attributes (TC intensity, translation (travel) speed and size). The sharper is the initial ocean temperature gradient, the stronger is the TC intensity (wind speed); the slower is the TC travelling speed and the larger is the TC size, the stronger is the OCE that is induced^31^,^33^. In this study, we use the 3D Price–Weller–Pinkel (3DPWP) ocean mixed layer model^33^ to estimate the OCE at each grid in the domain and over each of the 22 ensemble ocean fields. As the purpose here is to assess the change in the OCE due to the change in the initial ocean environment under global warming, the TC attributes used to calculate the OCE are fixed and the impact from the change in the ocean environment can thus be identified without co-varying factors.

As TC attributes have to be fixed and the wind speed in the CMIP5 TC is significantly underestimated due to coarse resolution^11^,^14^, CMIP5 TC data are not employed. Instead, we investigate 15 TC scenarios based on combinations of five TC intensity categories (categories 1–5, that is, weak to intense) and three TC travelling speeds (3, 5 and 7 m s^−1^, that is, slow-, moderate- and fast moving; Supplementary Table 2). These 15 TC scenarios entail a large spectrum of possible TC conditions to systematically assess the associated OCE.

The 3DPWP^33^ is a well-known ocean mixed layer model that is designed for calculating the OCE induced by a TC (refer to the Methods for details). For each scenario and at each grid, the 3DPWP is independently run with updated initial CMIP5 ocean profile input from 2006 to 2100 (95 years) with uniform TC forcing (same intensity, travelling speed, size and no track variability in grids). As TC forcing is fixed for each scenario, the change in OCE is only from influenced by the change in the initial ocean stratification; it is not affected by the change in TC attributes (because no change in TC attributes). Although the change in TC attributes in global warming may further modify the change in OCE, it is not discussed in this study because the objective here is the change in the OCE due to future ocean environmental changes.

On the basis of the above, the OCE is systematically calculated at each CMIP5 model grid, for each of the 22 CMIP5 ocean fields, for each year from 2006 to 2100, and for each of the 15 scenarios over the WNP and the NA. We believe this assessment is the most systematic and comprehensive OCE change assessment due to available data on future ocean conditions.

**Figure 2:**
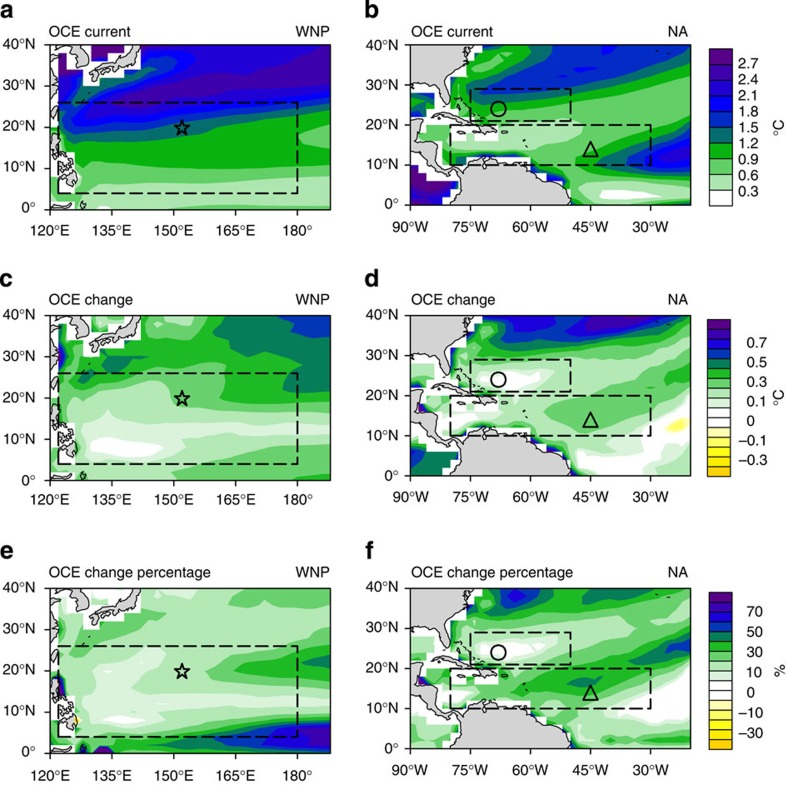
OCE simulated by the 3DPWP model under scenario 8. (**a**,**c**,**e**) The WNP and (**b**,**d**,**f**) the NA. (**a**,**b**) Existing OCE (based on initial profiles from the 2006–2015 average), (**c**,**d**) Changes in OCE due to global warming (that is, future OCE minus existing OCE; future OCE is based on initial profiles from the 2091–2100 average), (**e**,**f**) percentage change in OCE with respect to existing. The OCE changes for the remaining 14 scenarios are shown in Supplementary Figs 7 and 8. The two large boxes denote the MDRs over the WNP and the NA. The NA warm belt region is denoted by a smaller box (northern box) in **b**,**d**,**f**. The star, triangle and circle denote the positions of the selected representative stations for the three regions.

The results based on scenario 8 are illustrated in Fig. 2. This scenario is a moderate TC scenario for a category-3 TC intensity and 5 m s^−1^ travelling speed (Supplementary Table 2). The results of other scenarios are shown in Supplementary Figs 7 and 8 and Supplementary Tables 3 and 4. As shown in Fig. 2c–f, a corresponding increase in the OCE of ∼0.2–0.6 °C (∼10–30% enhancement) over most part of the WNP and NA is observed due to changes in the future ocean environment, with the exception of some localized regions (for example, the NA warm belt, Fig. 2d,f). As shown in Figs 1f,h and 2d, minimal OCE enhancement is observed over the NA warm belt region compared with NA MDR (0.056 versus 0.304 °C). These results are very robust among the ensemble members (Supplementary Fig. 9)

For the remaining 14 TC scenarios, consistent results of OCE enhancement under global warming are obtained, and a greater increase in the OCE for stronger and slower-moving TC scenarios is found (Supplementary Tables 2–4 and Supplementary Figs 7 and 8). As quantitatively summarized in Supplementary Tables 3 and 4, the future MME OCE mostly increases by ∼20% over the two MDRs with respect to the existing MME OCE. These data are also robust for the majority of the ensemble members (Supplementary Fig. 10). The same experiments were conducted using the CMIP2 initial profiles from Knutson *et al*.^2^. Owing to less stratification sharpening in the CMIP2 profiles, the OCE increase was only ∼40% of the increase observed in this study (Supplementary Tables 5–8, Supplementary Figs 5, 6, 11 and 12).

### Effect of OCE change and comparison with relative SST change

As introduced, the OCE is a TC's self-induced negative feedback due to the reduced during-TC SST and air–sea flux supply in the intensification^21^,^22^,^23^,^25^,^28^,^29^,^30^. The increase in OCE due to global warming suggests the possibility that this negative feedback may enhance in the future. To examine whether this OCE enhancement has an appreciable impact on future TC intensity, we compare it with a well-known empirical parameter related to TC activity—the relative SST^6^,^8^,^9^,^10^,^13^. Relative SST is defined as the local SST at each grid minus the remote tropical mean SST. Although the local SST warming promotes TC activity due to global warming^3^, this effect can be offset by the increase in the remote SST by warming the tropical atmosphere^6^. According to recent modelling studies, the trend of the relative SST is the key explanatory parameter for TC activity due to global warming instead of the trend of the local SST^6^,^8^,^9^,^10^,^13^.

**Figure 3:**
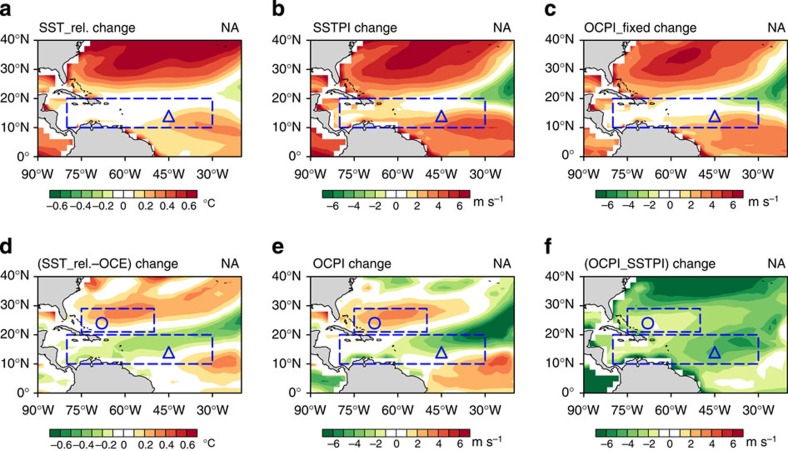
Trends in relative SST and PI under TC forcing scenario 8. (**a**) Relative SST change. (**b**) SST_PI change. (**c**) OC_PI change with fixed existing OCE. (**d**) Combined role of relative SST change and OCE change. (**e**) OC_PI change with increasing OCE under global warming. (**f**) Difference between OC_PI and SST_PI change. The WNP results are detailed in Supplementary Fig. 13. OCE and *T*_mix_ input to OC_PI are based on Fig. 2. The OC_PI changes and their differences from SST_PI change for the remaining 14 scenarios are shown in Supplementary Figs 15–18.

In Figs 2d and 3a, the magnitude of the trends for OCE and relative SST (both in °C) over the NA are comparable—∼0.1–0.6 °C (similar results obtained for the WNP, Fig. 2c and Supplementary Fig. 13)—suggests the potential importance of changes in future OCE on future TC activity. Next we examine the impact of OCE enhancement on potential intensity (PI), which is a key parameter for projecting future TC activity^6^,^8^,^10^,^13^,^30^,^34^. PI estimates the intensity upper bound of a TC based on the environmental atmospheric and ocean conditions^6^,^34^,^35^. As it can be efficiently calculated, it complements the expensive dynamical-downscaling projections^8^,^9^,^13^ in future TC projections because it can be applied across many ensemble members to assess the model-to-model dependence^6^.

Four experiments were conducted. Experiment 1 is the original PI (referred to as SST_PI in this study)^6^,^34^. The results show a positive trend of ∼0–6 m s^−1^ over the NA, which corresponds to the relative SST trend (Fig. 3a,b) noted by Vecchi and Soden^6^ and others^8^,^9^,^10^,^13^. However, SST_PI is an ‘uncoupled' PI without the ocean subsurface contribution or OCE because it only uses the pre-TC (undisturbed) SST and atmospheric conditions for estimation^6^,^34^. The TC–ocean system is an intrinsic coupled system; TCs do interact with the subsurface ocean in reality^22^,^23^,^24^,^25^,^26^,^27^,^31^. As a result, SST_PI can substantially overestimate the intensity upper bound^30^,^34^,^35^.

To address this limitation, Lin *et al*.^30^ proposed a revision named the Ocean Coupling PI (OC_PI). OC_PI replaces the pre-TC SST by the during-TC SST (refer to Methods) as the input, and thus OCE can be included. As the during-TC SST is the actual TC–ocean coupling SST that TCs encounter during intensification^31^,^33^, a more realistic intensity upper bound can be obtained^30^.

The remaining three experiments are coupled experiments based on OC_PI (that is, with the inclusion of the OCE). In experiment 2, OCE is fixed at the current amount (refer to Fig. 2a,b). In this experiment, no OCE enhancement in the future is assumed and the same amount of OCE is applied to both existing and future situations. In experiment 3, the OCE is allowed to increase due to global warming, that is, the existing OCE in Fig. 2a,b plus the OCE enhancement in Fig. 2c,d. Experiment 4 is similar to experiment 3 but the OCE only enhances by 40% due to global warming. This finding is an analogy to the results obtained by Knutson *et al*.^2^ (Supplementary Tables 5–8 and Figs 11 and 12).

As shown in Fig. 3b,c, the trends of experiment 1 (SST_PI) and experiment 2 (OC_PI with fixed OCE) are predominantly positive and similar, which indicates that if OCE is to be fixed at the present amount (that is, if no increase due to global warming), the trend in the coupled projection (that is, OC_PI) is similar to the trend for the uncoupled situation, which both reveal a positive trend. This result is consistent with the positive trend from the Emanuel^12^ coupling result, in which the ocean field is fixed at the present-day climatology.

**Figure 4:**
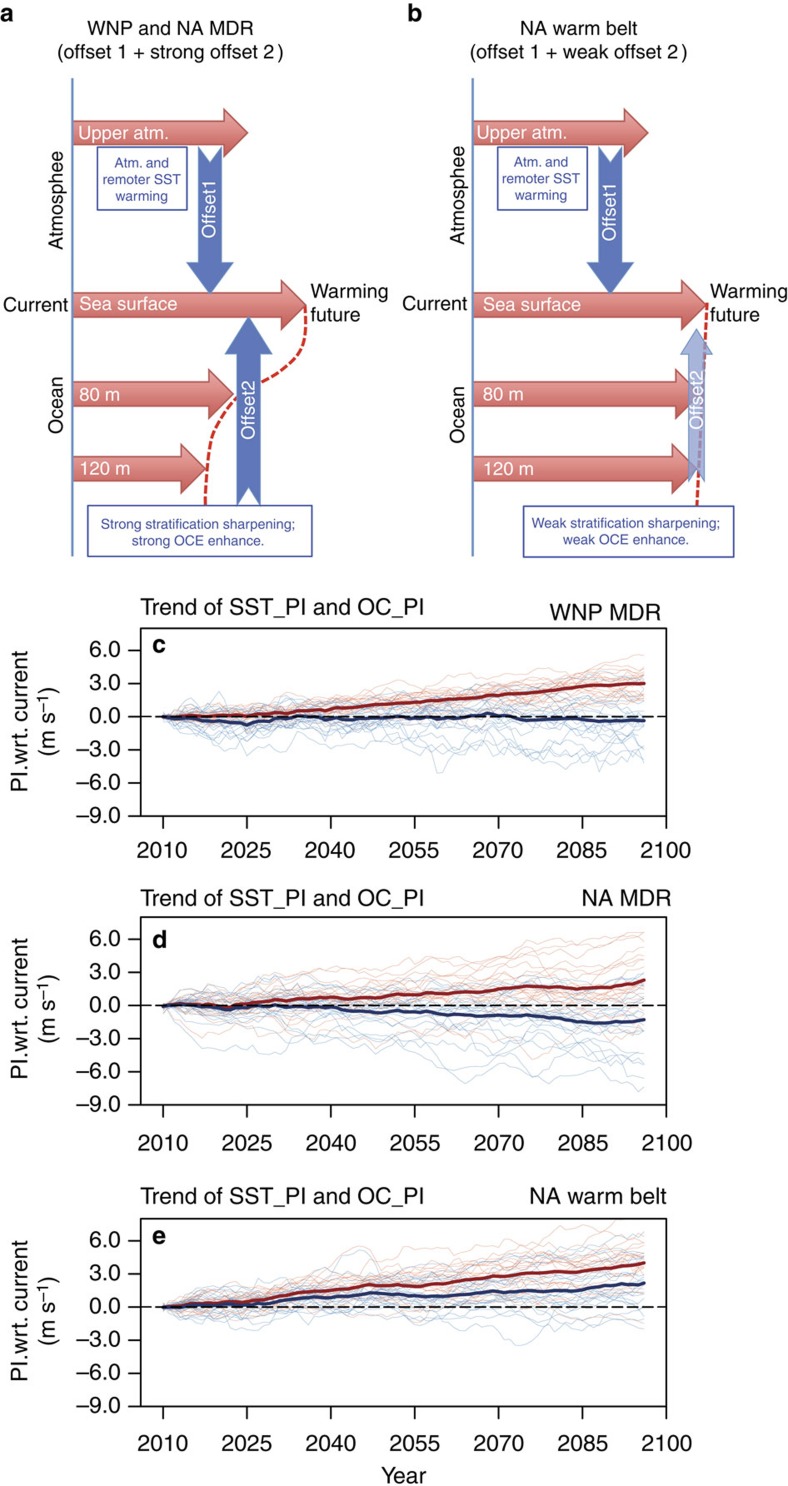
Schematic of two offsetting factors that can suppress future TC PI. Offset 1 is derived from the remote SST warming, while offset 2 is from the subsurface ocean thermal gradient sharpening and OCE enhancement. (**a**) Strong offset 2 is the situation over the MDRs over the WNP and the NA. (**b**) Weak offset 2 is the situation over the NA warm belt region. (**c**–**e**) The trends of SST_PI (red) and OC_PI (blue) for TC scenario 8 over the WNP MDR, the NA MDR and the NA warm belt. The thick (thin) curves represent the MME (22 individual member) results. Atm., atmosphere.

However, if the OCE is allowed to increase due to global warming (experiment 3), an approximate weakening in PI of 0–4 m s^−1^ is observed in the NA MDR (southern box in Fig. 3e,f) compared with the SST_PI (Fig. 3b). This finding suggests a possible suppressive effect due to an OCE enhancement to weaken the increasing SST_PI trend due to global warming over the NA MDR (Fig. 3f). Similar results are also observed in the WNP MDR (Fig. 4c, Supplementary Fig. 13f).

In experiment 4, the suppressive effect on the TC intensification from OCE enhancement remains visible; however, it is less evident than the suppressive effect in experiment 3 (refer to comparison between Fig. 3b,e and Supplementary Fig. 14b). As OCE only enhances by 40% in this experiment, the suppressive effect is weaker. This result also explains the relatively minor impact from the coupled experiment in Knutson *et al*.^2^.

Outside the MDR in the NA warm belt region, the suppressive effect is too weak and ineffective, as characterized by the evident net positive trend in OC_PI (northern box in Fig. 3e). As in Fig. 4e, the OC_PI trend (from experiment 3) is similar to the SST_PI trend, which is expected as the warm belt region exhibits minimal initial stratification sharpening (Fig. 1g,h) and OCE enhancement (Fig. 2d). The suppressive effect in this region is minimal under global warming.

Characterized by the positive OC_PI trend, this NA warm belt region (21–29° N, Fig. 3e) coincides with the ‘hot spot' region with an increased occurrence of category 4 and category 5 TCs from the dynamical-downscaling projections by Bender *et al*.^8^ and Knutson *et al*.^13^. The decreasing trend of OC_PI over the NA MDR (south of the warm belt, at 10–20° N, Fig. 3e) is also consistent with the decreasing trend in the dynamical-downscaling projections for category 4 and category 5 TCs^8^,^13^.

The OC_PI results for the remaining scenarios are consistent; they are detailed in Supplementary Figs 9 and 15–19. Typically, the suppressive effect (that is, difference between OC_PI and SST_PI) is stronger for a stronger TC intensity and slower TC travelling speed (due to stronger OCE enhancement; refer to Supplementary Figs 7 and 8).

## Discussion

Figure 4a,b summarizes the thermodynamic factors that impact future TC PI. Although local SST warming increases the PI due to global warming^3^,^34^, two offsetting factors weaken its positive impact. The first offsetting factor is derived from the atmospheric (and remote SST) warming mechanism (proposed by Vecchi and Soden^6^). The second offsetting factor is derived from the ocean subsurface stratification sharpening discussed in this study.

Owing to the more uniform warming of the tropical upper troposphere, the first offset works throughout the tropical storm basins^6^. However, the strength of the second offset is dependent on local initial ocean stratification conditions. Over large parts of the WNP and NA (including the two MDRs), the ocean subsurface effect can suppress TC PI due to the evident sharpening in the subsurface temperature gradient and enhance OCE (Figs 2c,d, 3f and 4a,c,d). Over certain localized regions, such as the NA warm belt, this second offset is too weak and ineffective, as the PI continues to increase due to global warming (Figs 3e and 4b,e).

This study suggests that a potentially important ocean subsurface negative effect (damper) that suppresses future TC intensification may occur in the NA and the WNP during global warming, even though it is not spatially uniform and may be weak in certain local regions. If so, future TC intensity projection (per cent increase of intensity) may not be as significant as projected by the uncoupled SST_PI projections^6^,^13^ over these regions.

## Methods

### Definition of key regions

The MDR of the WNP is defined as 4°–26° N, 122°–180° E. The MDR and the warm belt region of the NA are defined as 10°–20° N, 30°W–80° W and 21°–29° N, 50°W–75° W, respectively.

### Future and existing conditions

In this study, the future global warming condition is defined as the average of 2091–2100, whereas the existing condition is defined as the average of 2006–2015. A 9-year running mean is applied to the 95-year series (2100–2006) to remove the interannual variability and to delineate the signal from the long-term global warming impact.

### Ocean and atmosphere environmental fields from CMIP5

The 22 CMIP5 Global Climate Models include ACCESS1-0, ACCESS1-3, BCC-CSM1.1, CCSM4, CMCC-CM, CMCC-CMS, CMCC-CESM, CNRM-CM5, CSIRO-Mk3-6-0, FGOALS-g2, GFDL-CM3, HadGEM2-AO, IPSL-CM5A-LR, IPSL-CM5A-MR, IPSL-CM5B-LR, MIROC-ESM, MIROC-ESM-CHEM, MIROC5, MPI-ESM-LR, MPI-ESM-MR, MRI-CGCM3 and NorESM1-M (see http://pcmdi9.llnl.gov/, and http://cmip-pcmdi.llnl.gov/cmip5/availability.html). The CMIP5 ocean fields (that is, profile from SST to a depth of 1,000 m) are employed as the initial input to the 3DPWP model for the OCE calculations. The initial profile is updated each year based on the boreal TC season (July–October) average. The corresponding atmospheric environmental field provides the required atmospheric profile for each grid for the SST_PI and OC_PI calculations. Owing to an uneven original grid size and vertical resolution from each of the 22 ocean fields (Supplementary Table 1), the ocean horizontal and vertical fields are interpolated to regular grids and standard subsurface depths. The vertical resolutions of these models (Supplementary Table 1) have been significantly improved relative to the vertical resolutions of the models in previous studies^2^,^32^.

### MME and individual members

The atmospheric and oceanic environmental fields of all 22 CMIP5 models are interpolated into a horizontal 2° grid. The results (including OCE, SST_PI and OC_PI) are annually calculated for the TC season and for each CMIP5 model field, from 2006 to 2100. After obtaining the results for the individual models, the MME average is calculated to obtain the MME results.

### OCE calculation

The OCE is a function of initial ocean condition (profile) and TC parameters (including intensity, travelling speed and size)^31^,^33^. The sharper the subsurface thermal gradient (higher stratification), the stronger the wind speed, the slower the travelling speed, the larger the size and the stronger the OCE that is induced. In this study, we employ the 3DPWP ocean mixed layer model for the OCE calculations for each CMIP5 ocean grid^33^. The experimental design is summarized in the main text.

The 3DPWP model simulates the TC-induced OCE from two major mechanisms—vertical mixing and upwelling. It is a hydrostatic model with primitive equations of temperature, salinity and momentum^33^. It can solve for the wind-driven baroclinic ocean response to a TC and addresses turbulent vertical mixing in the upper ocean. The important process of vertical mixing in the 3DPWP model is implemented through the mixing parameterization. Density (determined by temperature and salinity) and velocity (driven by TC wind) of the upper ocean will be mixed vertically until three stability criteria are satisfied, which are static stability:


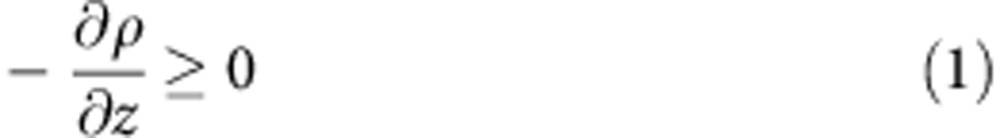


mixed-layer shear flow stability (bulk Richardson number *R*_b_):


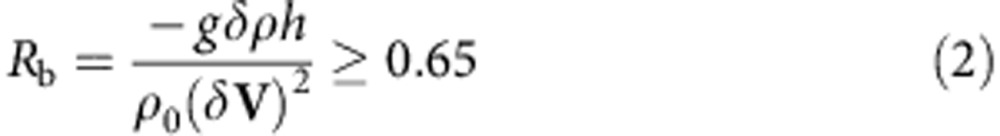


and stratified shear flow stability (gradient Richardson number *R*_g_):


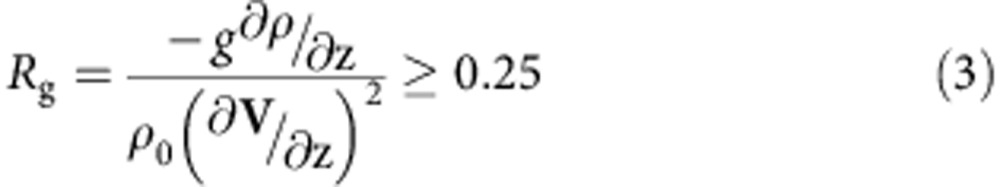


where **V** is the horizontal current, *ρ* is the density of sea water, *ρ*_0_ is the initial density, *z* is positive upward depth, *g* is the acceleration due to gravity and *δ* represents the vertical difference across the base of the mixed layer.

The ocean response to the TC is simulated in three spatial dimensions. Within each grid, the same initial ocean profile is specified. The horizontal resolution is 5 km; the vertical resolution is 5 m for the upper 100 m, 10 m for the upper 100–200 m and 50 m for the depth below 200 m. The input TC intensity (10-min maximum sustained surface wind speed) and translation speed (*U*_h_) is derived from the 15 scenarios (Supplementary Table 2).

For each scenario, the TC forcing (that is, intensity and *U*_h_) is fixed and encompasses the 22 CMIP5 ocean fields. The drag coefficient (*C*_d_), which is based on Powell *et al*.^36^, is suitable for the TC (high wind) condition. As illustrated in Supplementary Fig. 20, the OCE is more pronounced at the right-rear side of the track during the TC–ocean interaction, whereas the front side of the TC is less perturbed. From the TC perspective, the OCE is not uniform beneath the TC. To obtain an averaged OCE condition underneath a TC at each grid, OCE is calculated based on area averaging within a 70-km radius region (shaded region in Supplementary Fig. 20b) for the WNP. This area is equivalent to ∼2.5 radius of maximum wind region from the TC centre, as 28 km was a commonly observed radius of maximum wind for WNP TCs during intensification^30^. For the NA, the OCE is averaged within a 75-km radius as the typical radius of maximum wind over the NA is usually larger (assuming 30 km here) than the typical radius of maximum wind over the WNP. The appropriateness of this area-averaged OCE is evaluated for actual TC cases using *in situ* aircraft Airborne EXpendable Bathy Thermographs measurements from the Impact of Typhoon on the Pacific field campaign^29^,^30^ in 2010. As in Supplementary Fig. 20d, the OCE and the during-TC coupling SST (*T*_mix_, that is, pre-TC SST minus OCE) can be realistically estimated. (See details in Supplementary Discussion).

### SST_PI and OC_PI

The original PI (SST_PI) is based on the pre-TC SST and the atmospheric temperature and humidity profile


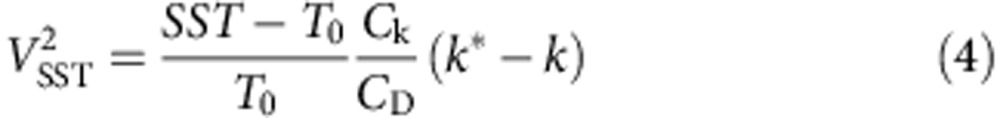


where *SST* denotes the pre-TC SST, *T*_0_ is the temperature of outflow, *C*_k_ is the enthalpy exchange coefficient, *C*_D_ is the drag coefficient, k* is the saturation enthalpy of the sea surface and *k* is the surface enthalpy in the TC environment^34^. It is calculated based on ref. 34 (programme from ftp://texmex.mit.edu/pub/emanuel/TCMAX/pcmin_2013.f). For OC_PI, the atmospheric inputs remain identical as SST_PI but the SST is replaced by the during-TC SST (*T*_mix_), which is estimated by the during-TC area-average output from the 3DPWP model (Supplementary Fig. 20b):


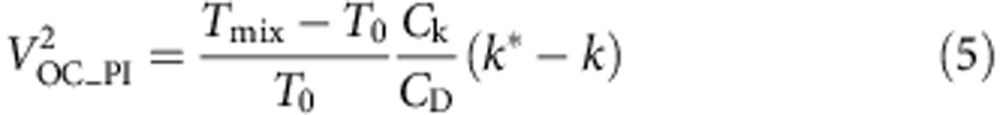


### Relative SST change

The relative SST change is calculated based on the SST change for each grid minus the tropical mean SST change (averaged from 30° S to 30° N). The boreal TC season (July–October) average for each year from 2006 to 2100 is employed.

### Discussions on the methods

The method employed in this research can be understood as a kind of downscaling method. The advantages of this method are that OCE change under a wide spectrum of TC conditions can be assessed, and not limited by the weak TC winds in CMIP5. It can also be efficiently applied to a large amount of CMIP5 models (22 here) to decrease the uncertainty due to possible model dependence. Finally, the concise framework of this method enables easy discussions on the relative role of ocean and atmosphere environment change on future TC PI. The disadvantages of this approach are that there is no track information and uniform TC parameters are applied throughout. In other words, it lacks the varying TC tracks and OCE is assessed gird by grid independently. Further details, including comparison with other methods, are given in the Supplementary Discussion.

## Additional information

**How to cite this article:** Huang, P. *et al*. Change in ocean subsurface environment to suppress tropical cyclone intensification under global warming. *Nat. Commun.* 6:7188 doi: 10.1038/ncomms8188 (2015).

## Supplementary Material

Supplementary Information Supplementary Figures 1-20, Supplementary Tables 1-8, Supplementary Discussion and Supplementary References
