# Supplementary material for: Change in ocean subsurface environment to suppress tropical cyclone intensification under global warming
Source: Nat Commun. 2015 May 18;6:7188. doi: 10.1038/ncomms8188 (PMC4479036; doi:10.1038/ncomms8188)
Supplement: Supplementary Information — Supplementary Figures 1-20, Supplementary Tables 1-8, Supplementary Discussion and Supplementary References [file ncomms8188-s1.pdf]

Supplementary Figures:

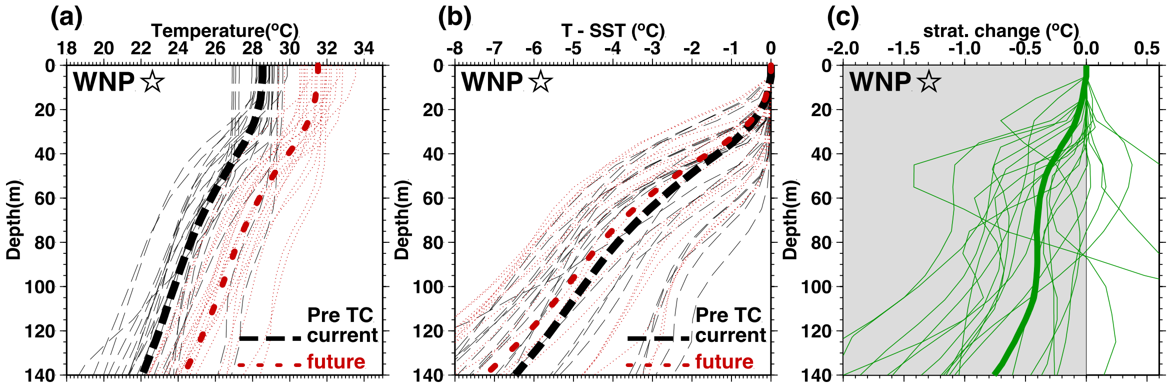

Supplementary Figure 1. **(a)** Current (black) and future (red) initial ocean thermal profiles at the representative station in the WNP MDR (location see star in Fig. 1a of the main text). The thick profiles are from MME and the thin profiles are from the 22 ensemble members. **(b)** The corresponding subsurface ocean stratification (i.e. subsurface temperature with respect to (wrt) SST). It can be seen that future stratification is sharper than current. In other words, though current subsurface ocean is colder than current SST, future subsurface ocean will be even colder than future SST. This is found not only in MME, but also in most of the 22 ensemble members. **(c)** The difference between future and current stratification (i.e. subtracting the future and current stratification in (b)). Therefore, profiles under negative x-axis region (grey-shaded region) are sharper stratification in future (wrt current). It can be seen that MME and most of the ensemble members are showing such stratification sharpening in the future.

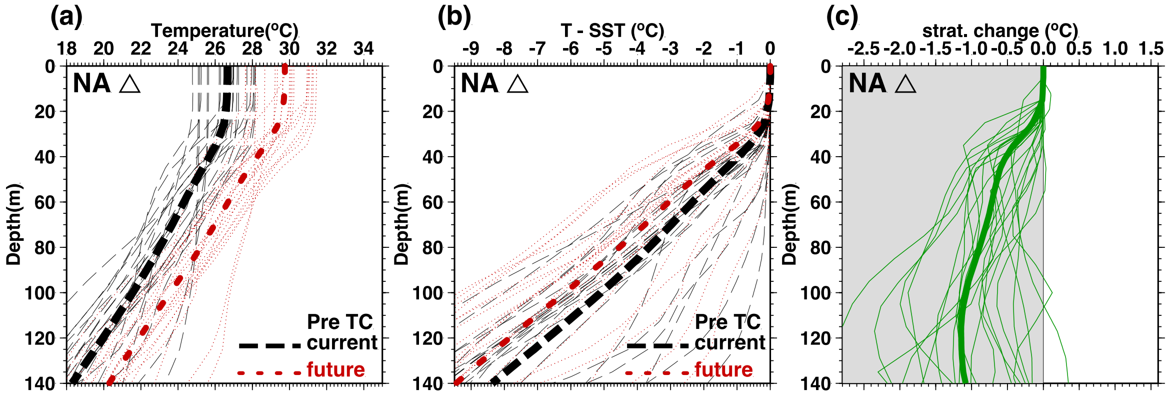

Supplementary Figure 2. (a) Current (black) and future (red) initial ocean thermal profiles at the representative station in the NA MDR (location see triangle in Fig. 1b of the main text) The thick profiles are from MME and the thin profiles are from the 22 ensemble members. (b) Subsurface ocean stratification, i.e., subsurface temperature at each depth with respect to SST. (c) The difference between future and current subsurface stratification.

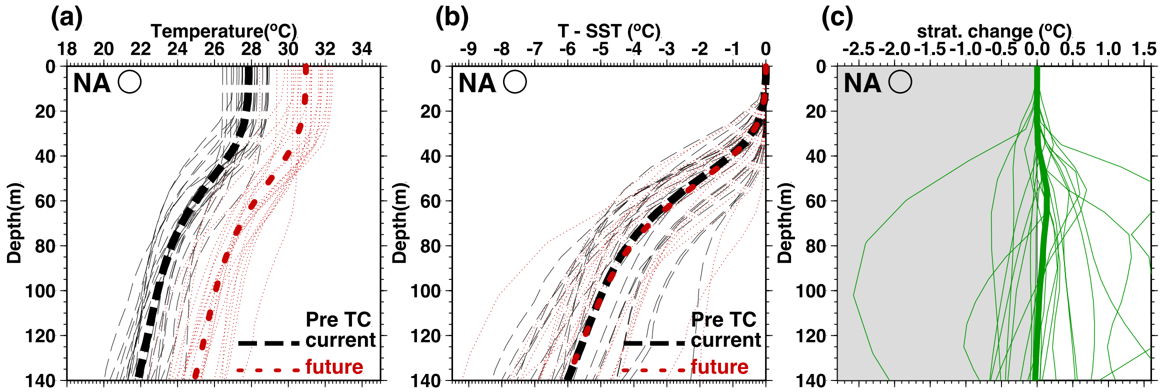

Supplementary Figure 3. (a) Current (black) and future (red) initial ocean thermal profiles at the representative station in the NA warm belt (location see circle in Fig. 1b of the main text) The thick profiles are from MME and the thin profiles are from the 22 ensemble members. (b) Subsurface ocean stratification, i.e., subsurface temperature at each depth with respect to SST. (c) The difference between future and current subsurface stratification.

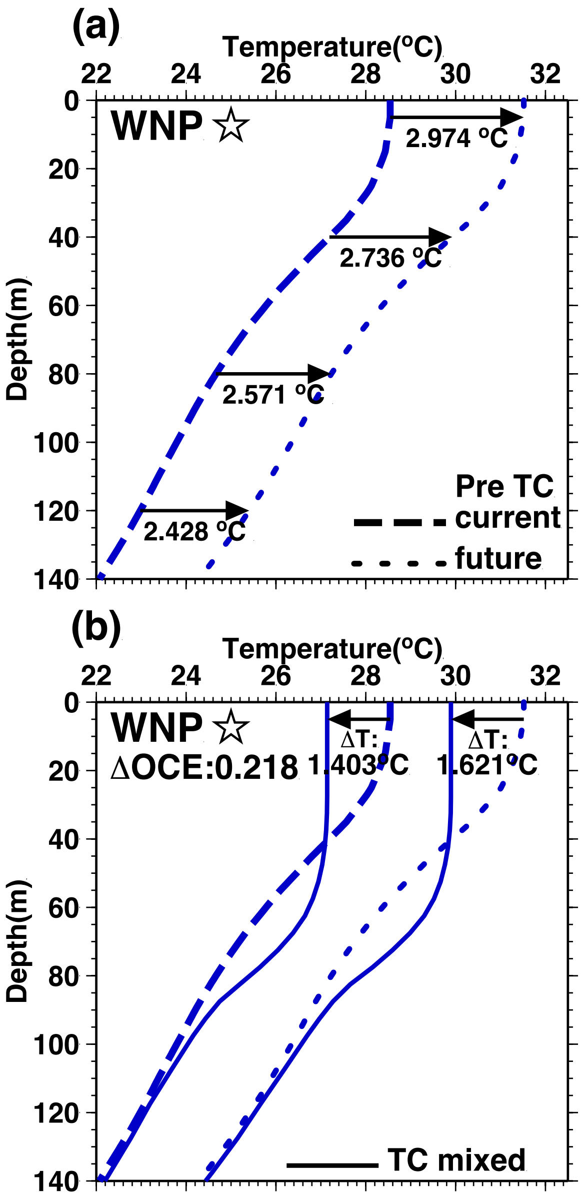

Supplementary Figure 4. **(a)** Comparison of current (2006–2014 average) and future (2092–2100 average) initial MME ocean thermal profiles at a representative station (152°E, 20°N) in the WNP MDR (location illustrated as star in Fig. 1a). Sharpening of ocean subsurface gradient in the future is visible. **(b)** Using profiles in (a) as initial input, the 3DPWP simulation of current OCE and future OCE under a moderate TC scenario (scenario 8). Due to stratification sharpening in the future initial ocean profile, future OCE (1.621 °C) is stronger than current OCE (1.403 °C) by 0.218 °C, i.e., ~ 16% wrt current OCE.

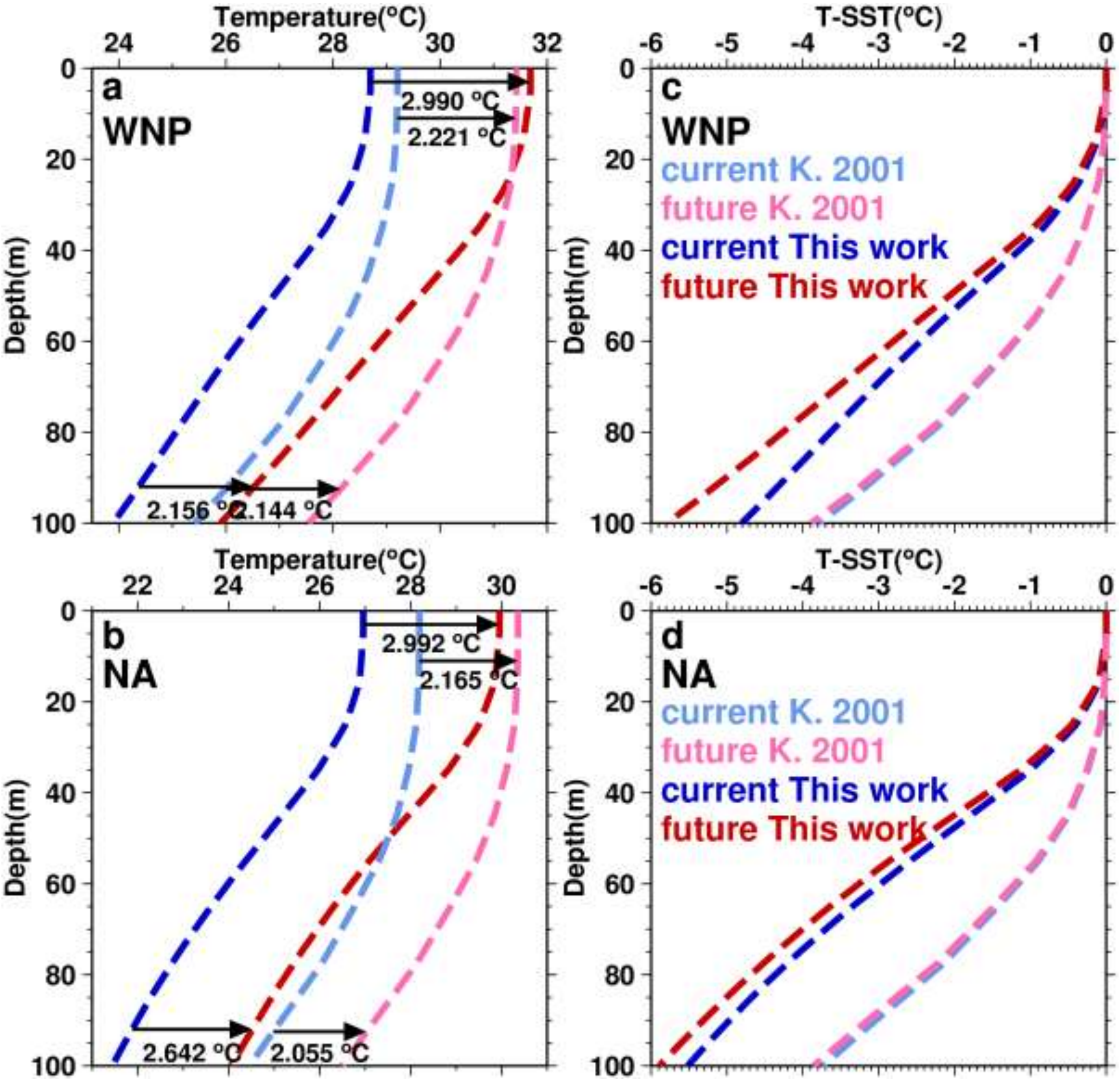

Supplementary Figure 5. (a,b) Comparison between the initial pre-TC profiles between this work (22 CMIP5 model MME) and Knutson et al. 2001 (based on CMIP2) over WNP and NA MDRs. (c,d) as in (a,b), but for the subsurface ocean stratification (i.e. subsurface temperature wrt SST.). It can be seen that subsurface thermal gradient sharpening (a,b) and stratification increase in the future (c,d) are weaker in the Knutson et al. 2001 profiles.

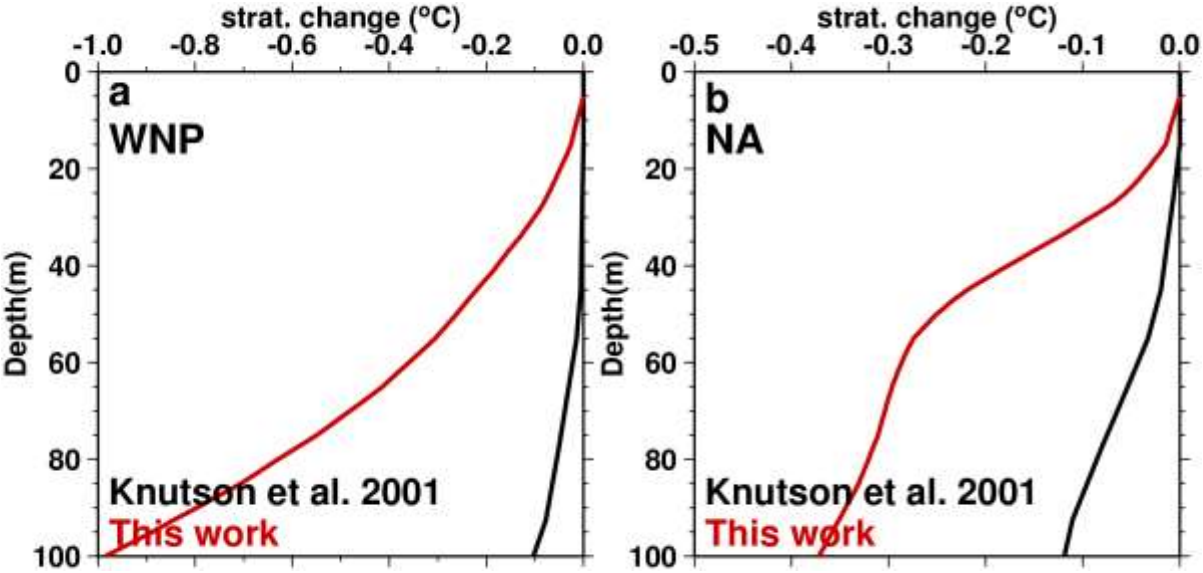

Supplementary Figure 6. Comparison between this work and Knutson et al. 2001, based on pre-TC ocean subsurface stratification change (future minus current) for WNP MDR (a) and NA MDR (b).

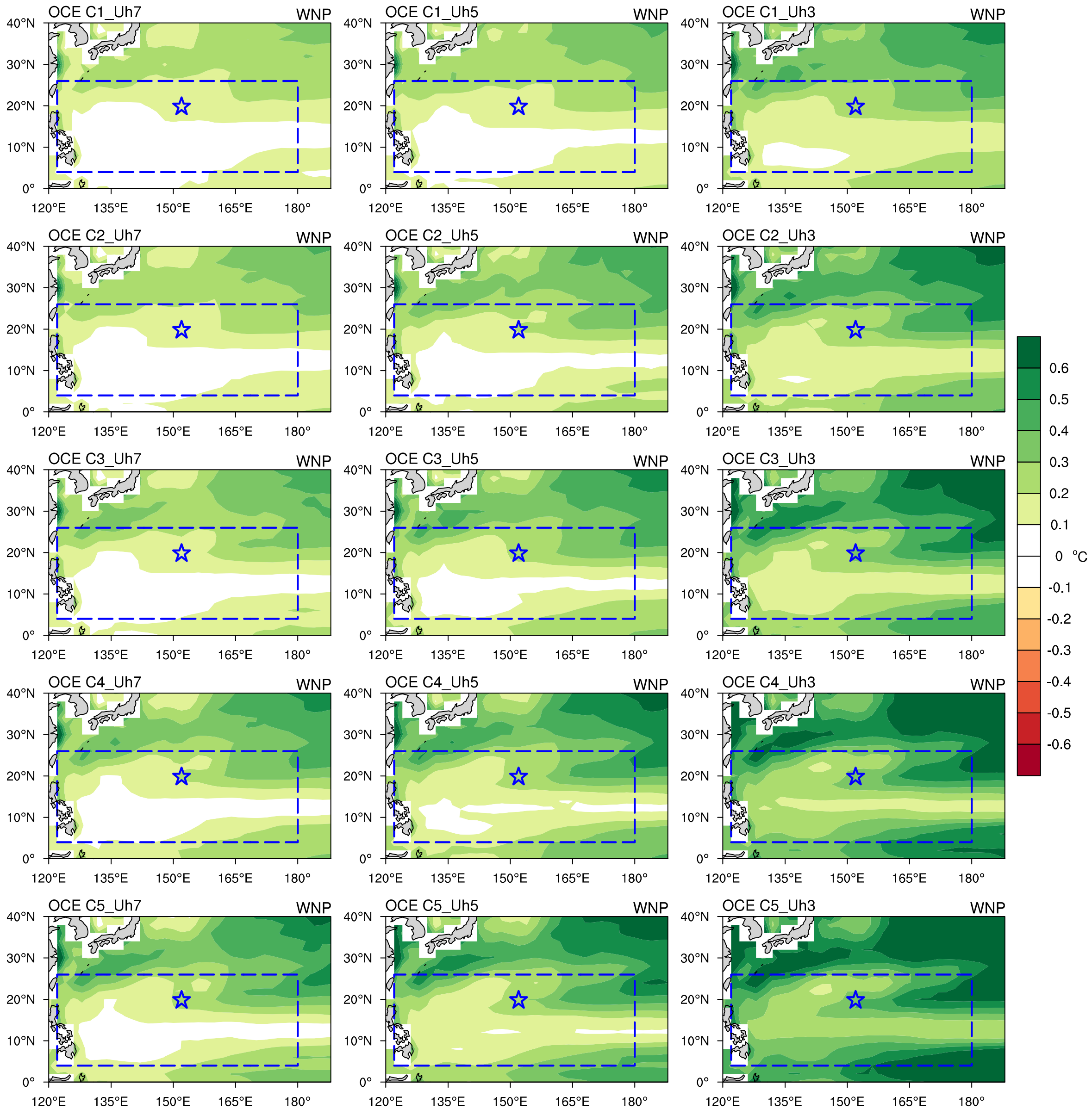

Supplementary Figure 7. OCE change over the WNP, based on the 15 TC forcing scenarios. Green colour indicates increase (enhance) in the TC-induced OCE.

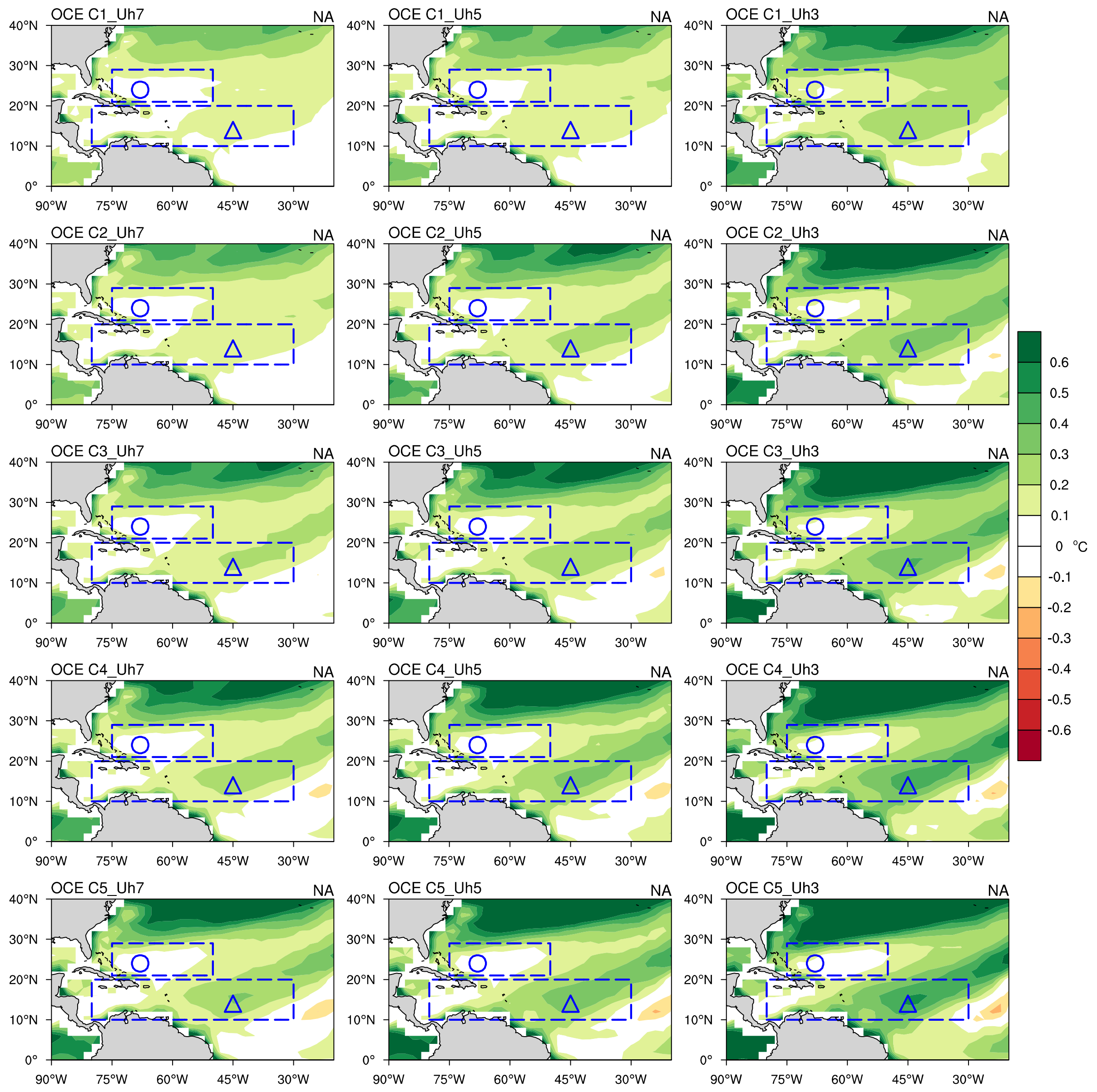

Supplementary Figure 8. OCE change over the NA, based on the 15 TC forcing scenarios. Green colour indicates increase (enhance) in the TC-induced OCE.

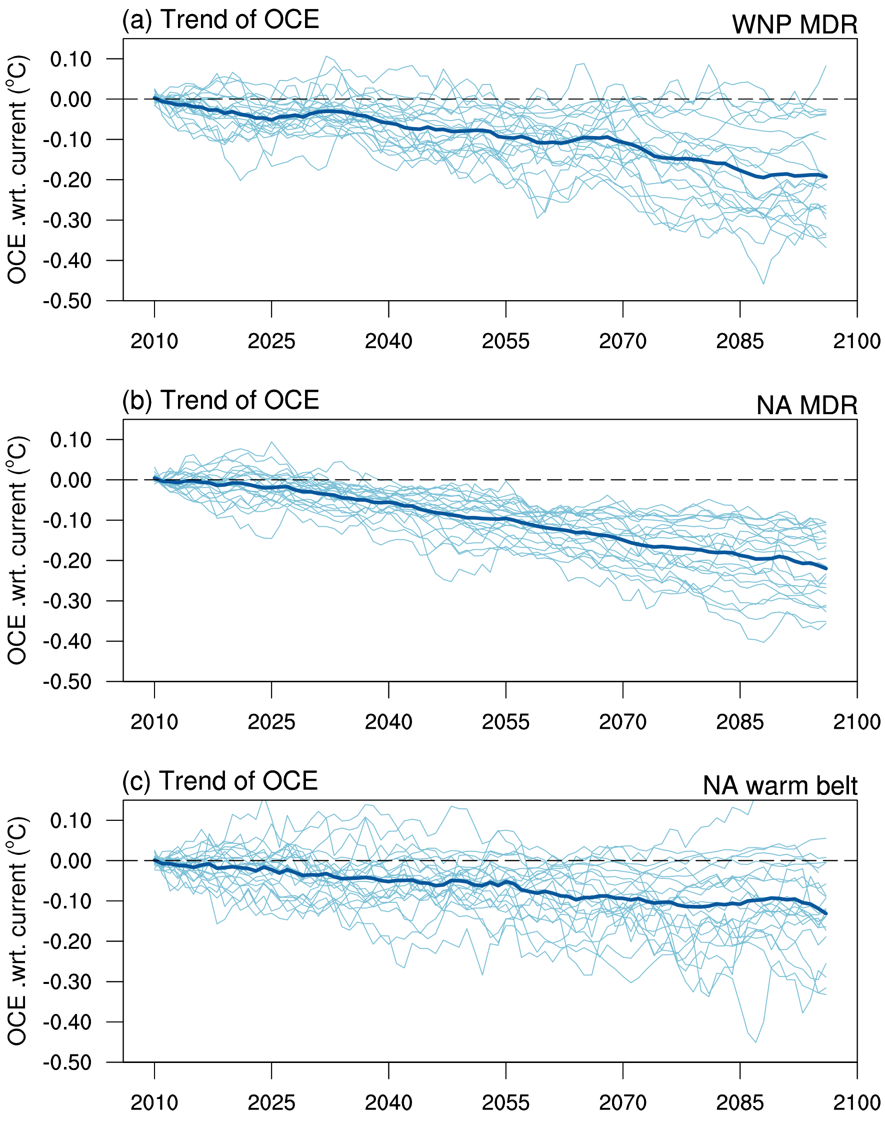

Supplementary Figure 9. The trends of OCE for TC scenario 8 over the WNP main development region, the NA main development region, and the NA warm belt. The thick (thin) curves represent the multi-model ensemble (22 individual member) results.

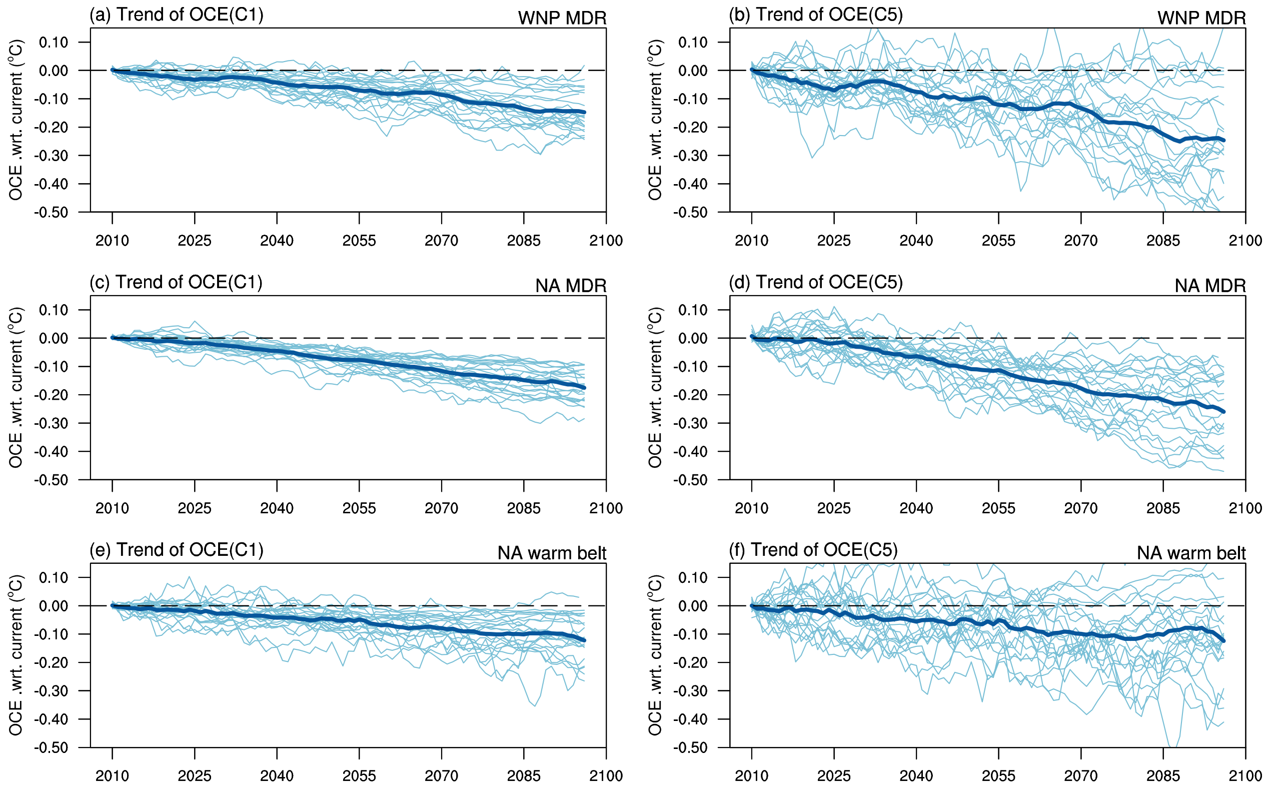

Supplementary Figure 10. (a, c, e) The trends of OCE for TC scenario 6 (category 1 intensity and 5 m s^{-1} TC translation speed) over the WNP main development region, the NA main development region, and the NA warm belt. (b, d, f) As in (a,c,e) but under TC forcing scenario 10 (category 5 intensity and 5 m s^{-1} TC translation speed).

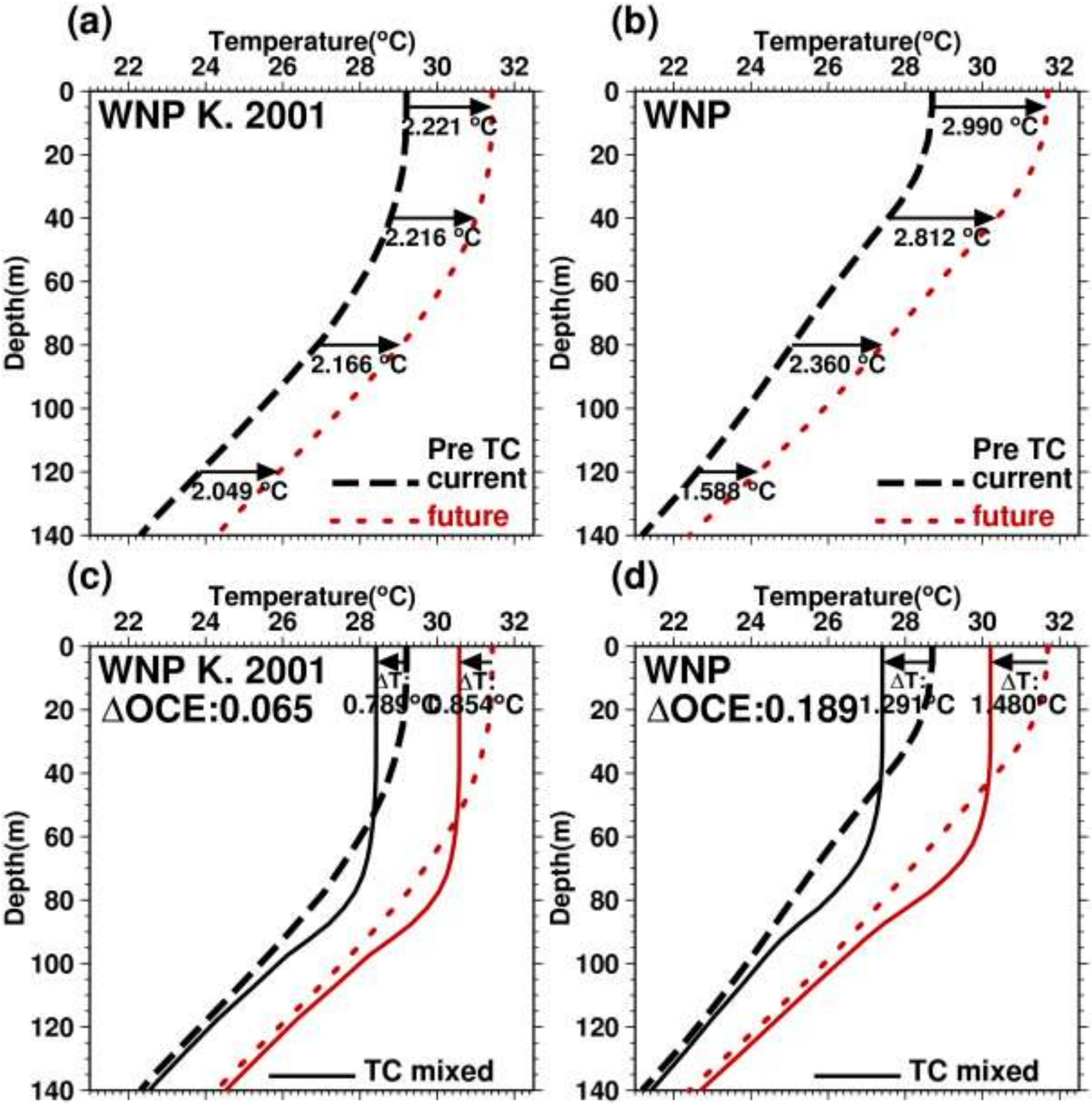

Supplementary Figure 11. Comparison of TC-induced OCE based on the initial, pre-TC profiles from Knutson et al. 2001 and this work, over the WNP MDR. (a,b) The pre-TC initial profile comparison. (c,d) OCE results comparison. 3DPWP was run under scenario 8 (i.e. category 3 intensity and 5 m s^{-1} translation speed). For other scenarios, see Supplementary Tables 5 and 7.

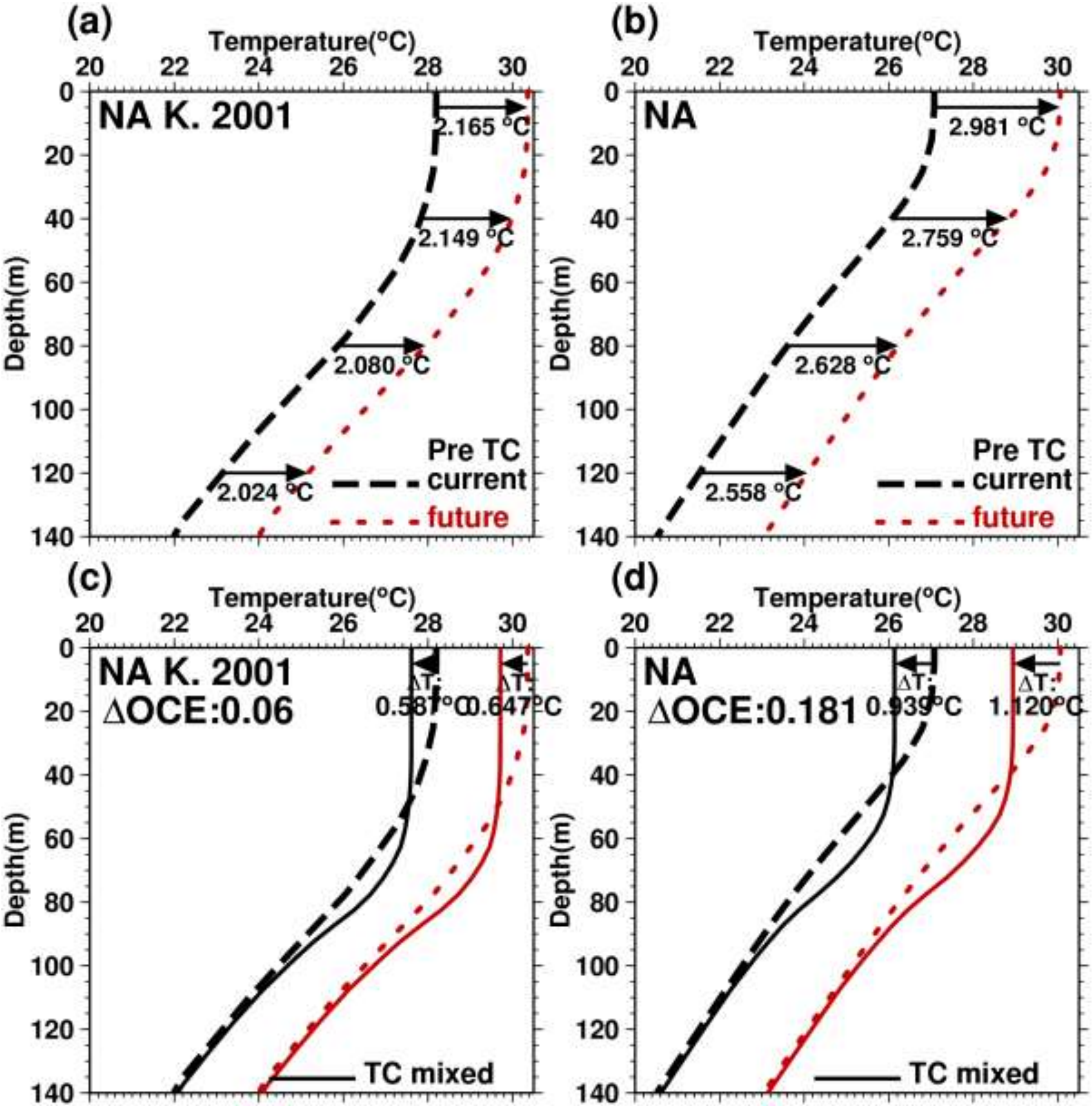

Supplementary Figure 12. Comparison of TC-induced OCE based on the initial, pre-TC profiles from Knutson et al. 2001 and this work, over the NA MDR. (a,b) The pre-TC initial profile comparison. (c,d) OCE results comparison. 3DPWP was run under scenario 8 (i.e. category 3 intensity and 5 m s^{-1} translation speed). For other scenarios, see Supplementary Tables 6 and 8.

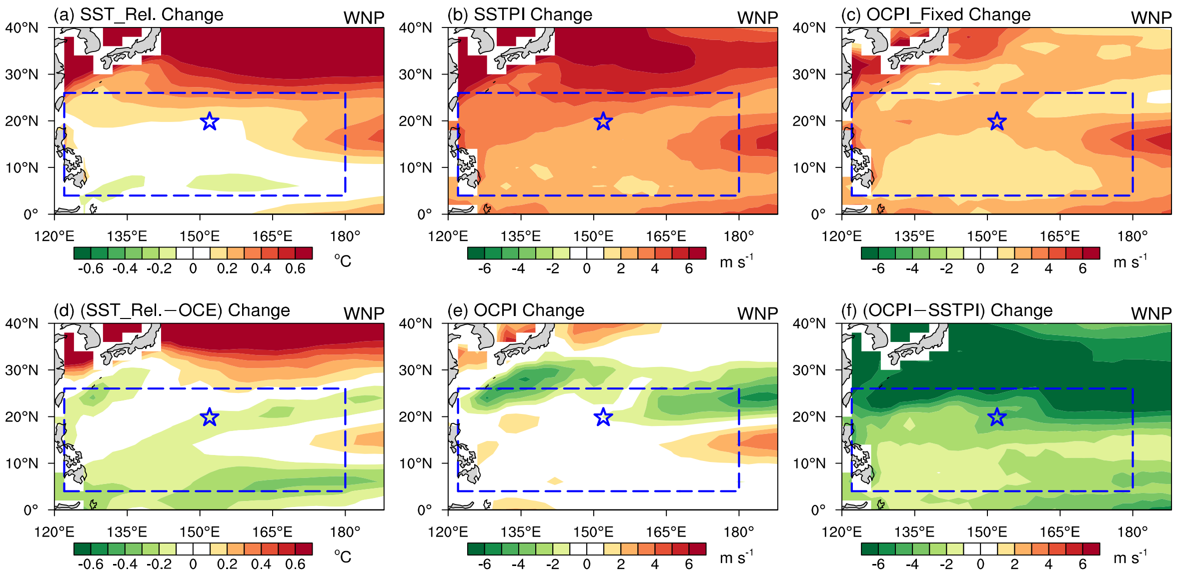

Supplementary Figure 13. Trends (changes) of relative SST and TC Potential Intensity (PI) in the WNP. (a) Relative SST change. (b) SST_PI change. (c) OC_PI change with fixed existing OCE. (d) Combined role of relative SST change and OCE change. (e) OC_PI change with increasing OCE under global warming. (f) Difference between OC_PI (from (d)) and SST_PI change. The OC_PI changes (global warming scenario) and their differences from SST_PI change for the remaining 14 scenarios are shown in Supplementary Figs. 15–18.

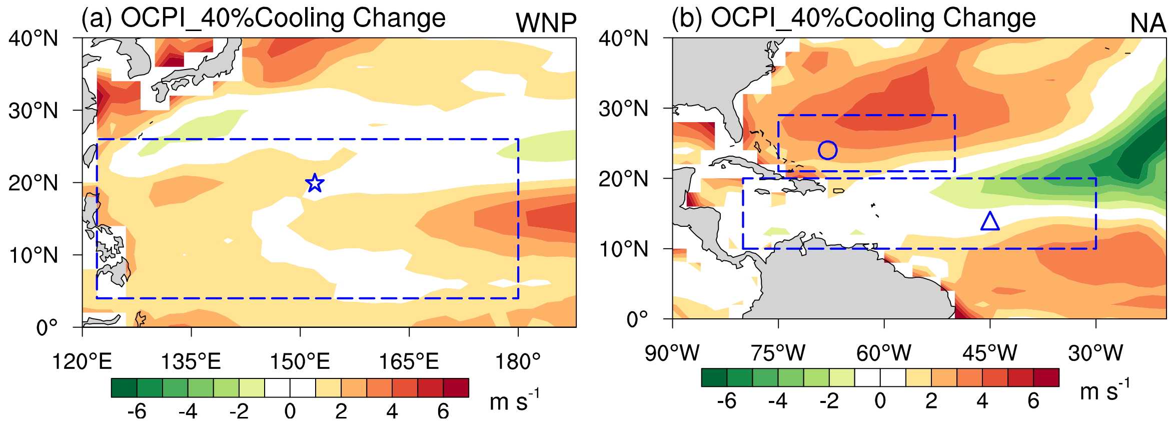

Supplementary Figure 14. OC_PI change with 40% of OCE increase in future, an analogy to the Knutson et al. 2001 situation.

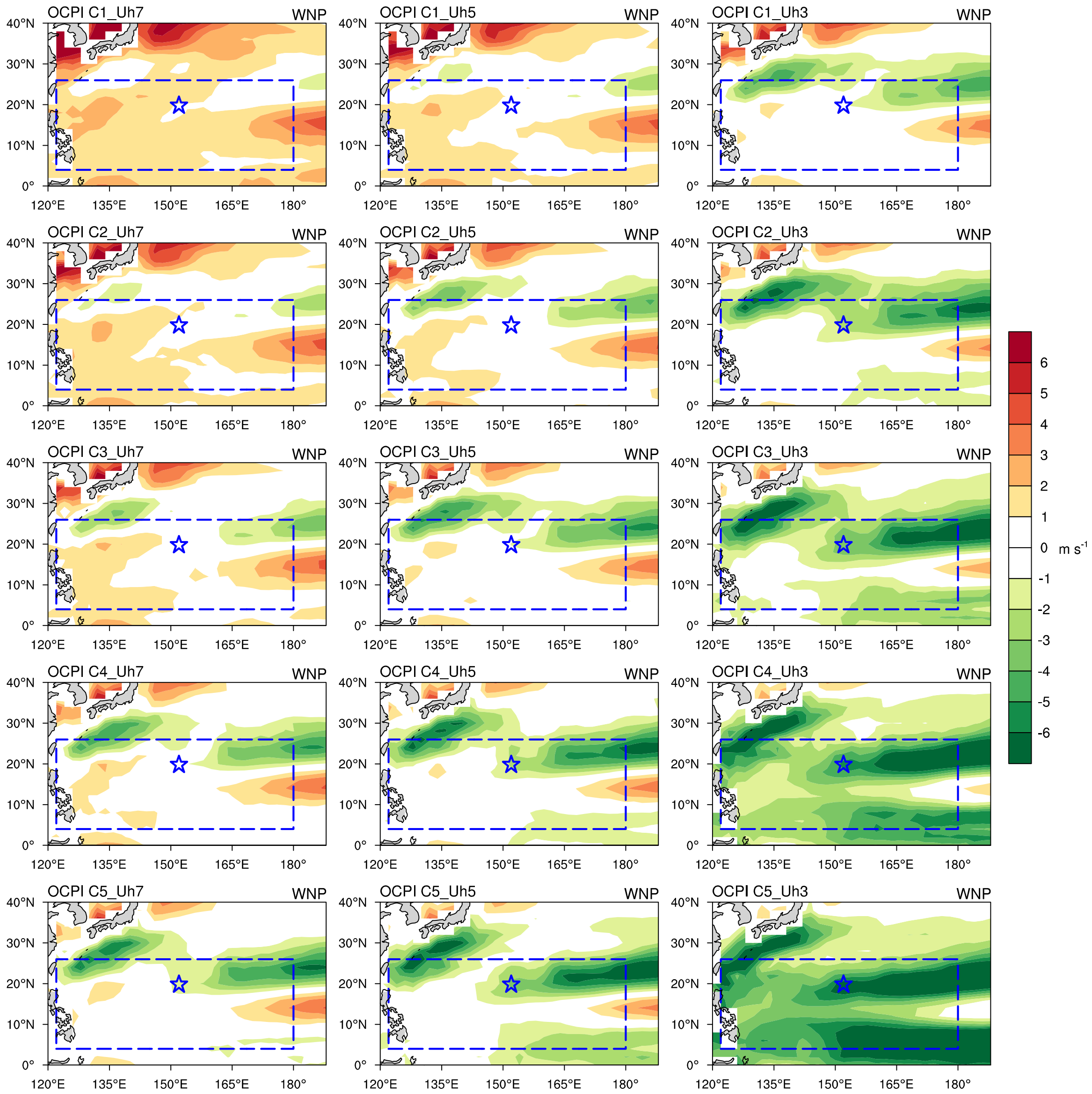

Supplementary Figure 15. OC_PI change over the WNP based on the 15 TC forcing scenarios.

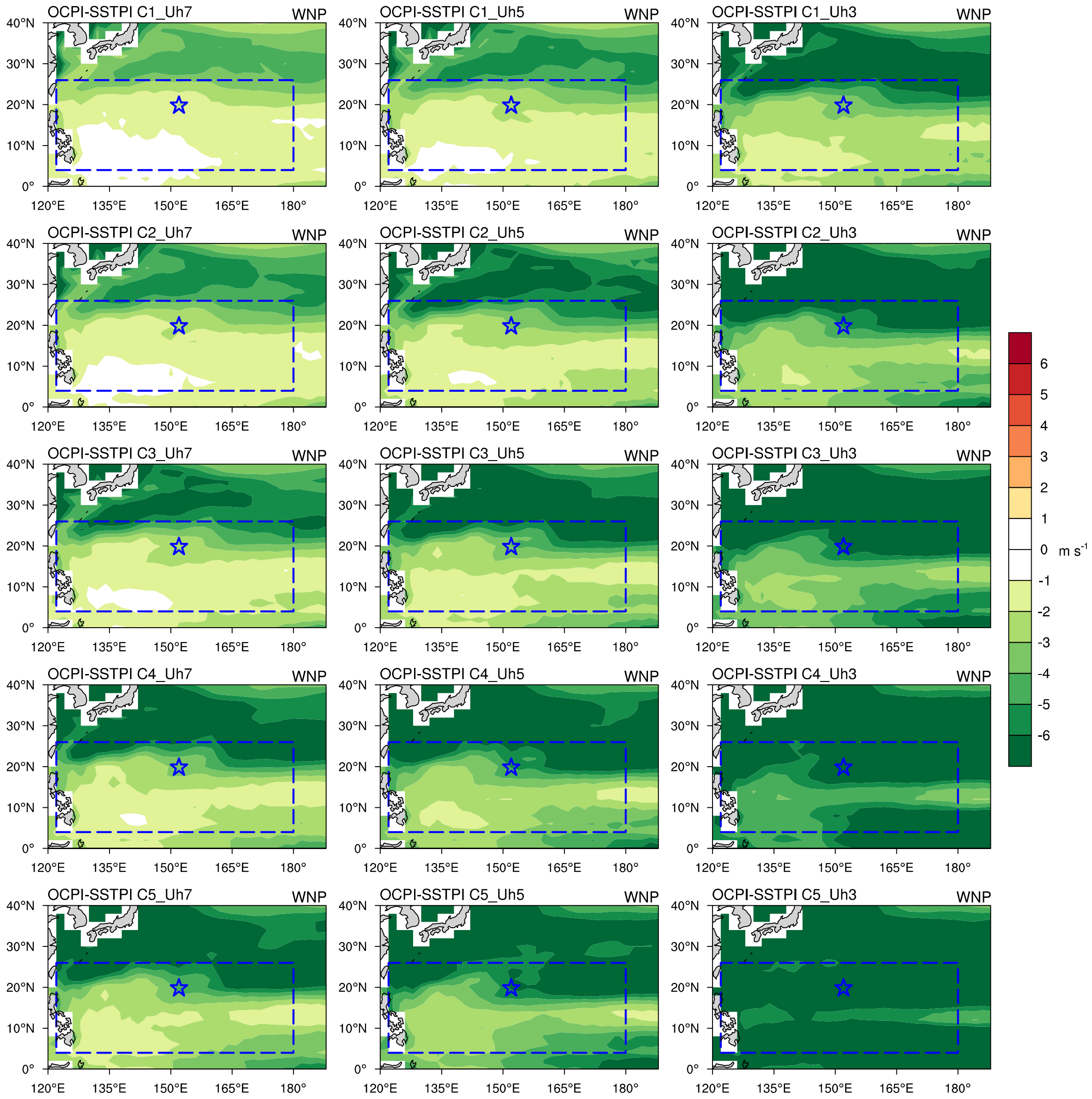

Supplementary Figure 16. Difference between OC_PI change and SST_PI change over the WNP for the 15 TC forcing scenarios.

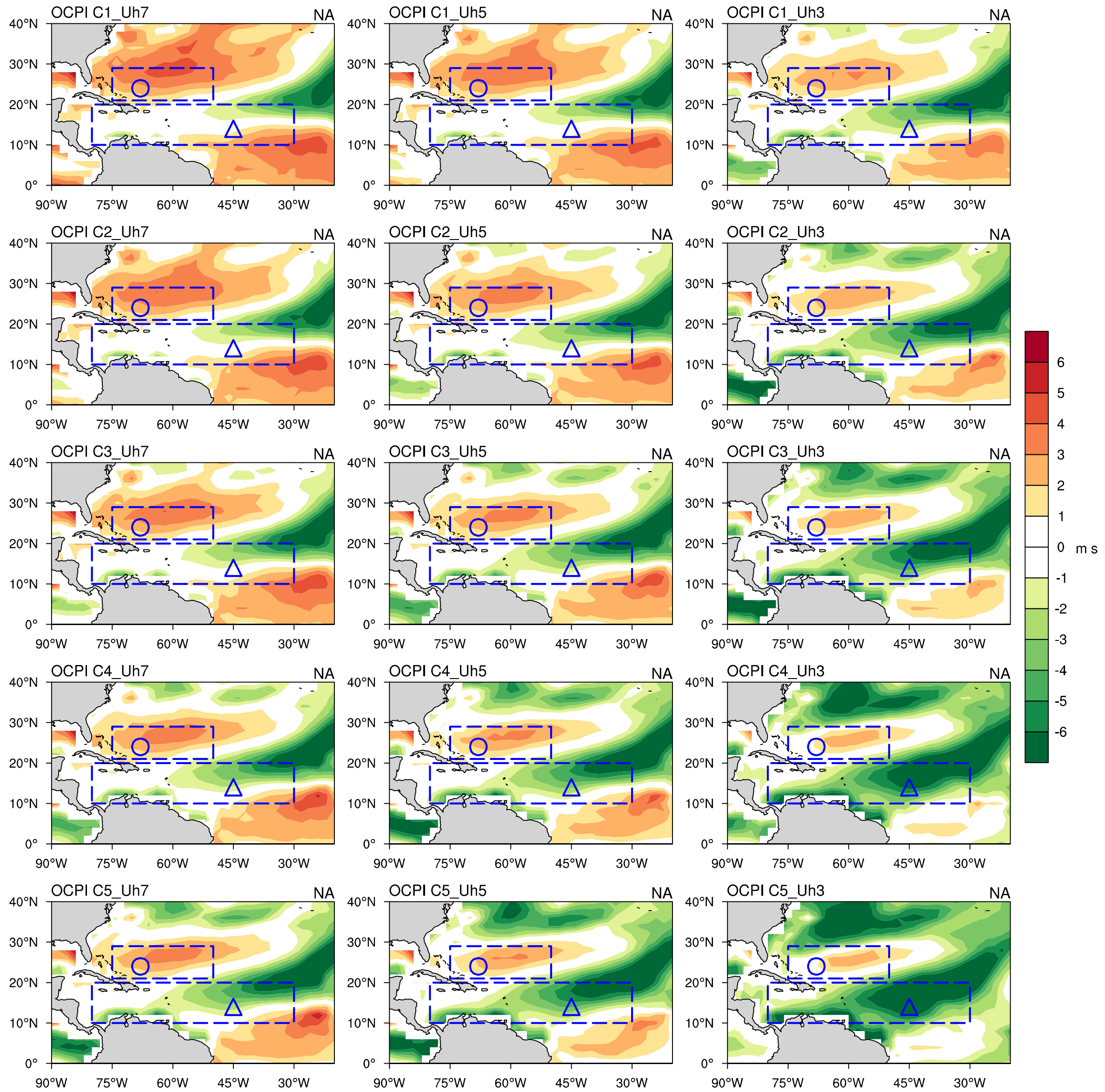

Supplementary Figure 17. OC_PI change over the NA based on the 15 TC forcing scenarios.

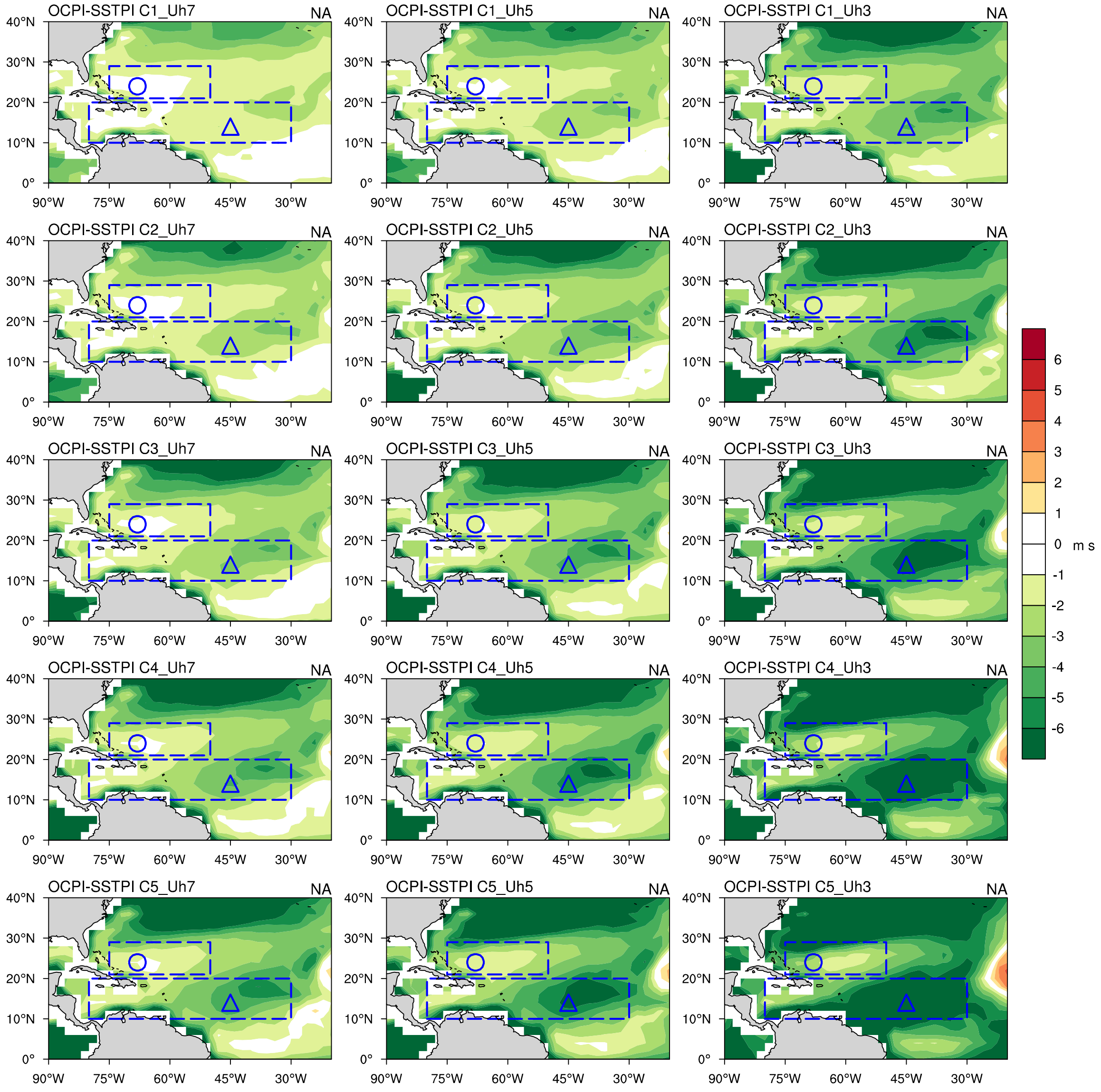

Supplementary Figure 18. Difference between OC_PI change and SST_PI change over the NA for the 15 TC forcing scenarios.

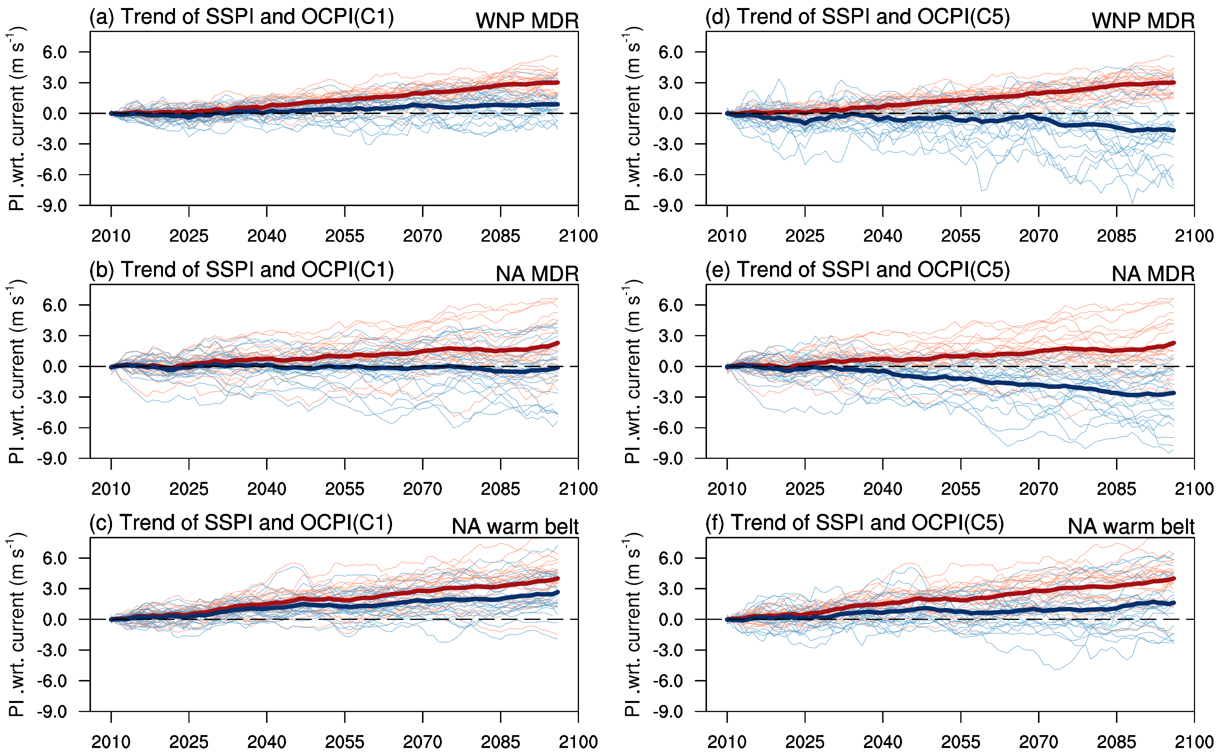

Supplementary Figure 19. (a, b, c) The trends of SST_PI (red) and OC_PI (blue) for TC scenario 6 (category 1 intensity and 5 m s^{-1} TC translation speed) over the WNP main development region, the NA main development region, and the NA warm belt. (d, e, f) As in (a,b,c) but under TC forcing scenario 10 (category 5 intensity and 5 m s^{-1} TC translation speed).

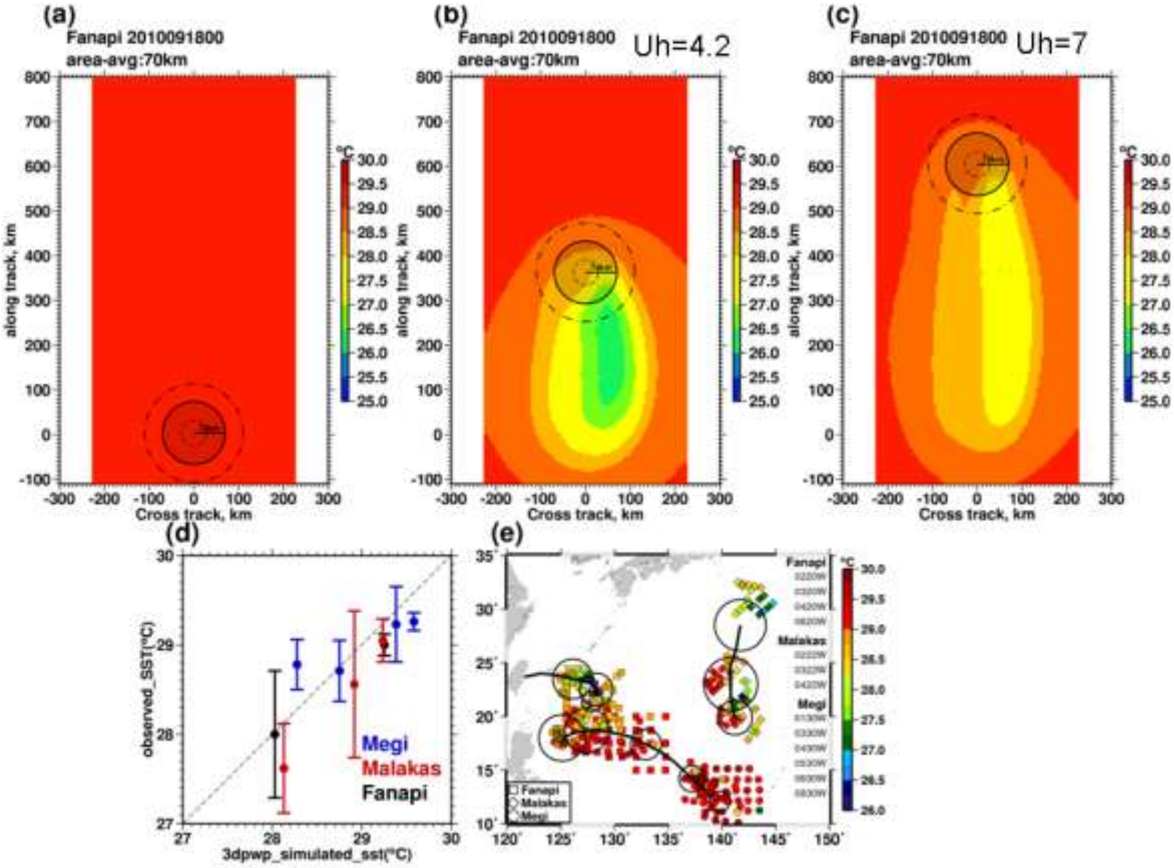

Supplementary Figure 20. An illustration of OCE simulation from the 3DPWP model at a grid, showing (a) pre-TC SST, and (b,c) during-TC SST distribution under TC translation speed (Uh) of 4.2 ms^{-1} (b) and 7 ms^{-1} (c). TC is moving from south to north at a constant translation speed in (b, c). Note that for each run, the intensity, structure and Uh of TC do not change. It can be seen that OCE is more pronounced at the right-rear side of the track. The OCE result of this research is based on area-averaging from the TC centre (i.e. 70km radius for WNP and 75km radius for NA). (d) Validation of the 3DPWP OCE simulation (x-axis). The observed SST (y axis, i.e., *in situ* TC-ocean coupling SST or Tmix) was from the average of more than 400 US C130 Airborne AXBT measurements obtained during the ITOP (Impact of Typhoon on Pacific) field campaign in 2010. (e) The original during-TC AXBT observations from the 13 C130 flights during ITOP (after Lin *et al.* 2013[1]).

Supplementary Tables

Supplementary Table 1: Vertical resolution of the upper subsurface ocean in the 22 CMIP5 models.

Model Name	Levels (0~100m)	Levels (0~400m)	Model Name	Levels (0~100m)	Levels (0~400m)
**ACCESS1-0**	**11**	**26**	**HadGEM2-AO**	**11**	**22**
**ACCESS1-3**	**11**	**26**	**IPSL-CM5A-LR**	**11**	**20**
**BCC-CSM1-1**	**11**	**26**	**IPSL-CM5A-MR**	**11**	**20**
**CCSM4**	**11**	**31**	**IPSL-CM5B-LR**	**11**	**20**
**CMCC-CESM**	**11**	**20**	**MIROC-ESM-CHEM**	**14**	**21**
**CMCC-CM**	**11**	**20**	**MIROC-ESM**	**14**	**21**
**CMCC-CMS**	**11**	**20**	**MIROC5**	**12**	**22**
**CNRM-CM5**	**11**	**22**	**MPI-ESM-LR**	**10**	**17**
**CSIRO-Mk3-6-0**	**7**	**14**	**MPI-ESM-MR**	**10**	**17**
**FGOALS-g2**	**11**	**20**	**MRI-CGCM3**	**11**	**23**
**GFDL-CM3**	**11**	**30**	**NorESM1-M**	**13**	**25**

Supplementary Table 2: The 15 hypothetical TC scenarios, as combined from 5 TC intensity categories (in 1-min (10-min) maximum sustained surface wind speed from the Saffir-Simpson scale, y axis) and 3 TC travelling speeds (from fast to slow, x axis)

Scenario No.	**Uh=7 ms^{-1} (fast)**	**Uh=5 ms^{-1} (moderate)**	**Uh=3 ms^{-1} (slow)**
**Category 1 38 (35) m s^{-1} (weak)**	1	6	11
**Category 2 46 (42) m s^{-1}**	2	7	12
**Category 3 54 (49) m s^{-1} (moderate)**	3	8	13
**Category 4 64 (58) m s^{-1}**	4	9	14
**Category 5 72 (65) m s^{-1} (intense)**	5	10	15

Supplementary Table 3: Comparison of current (2006-2015) and future (2091-2100) MME OCE over the WNP MDR for the 15 TC scenarios. OCE trend (future minus current) and the percentage of OCE increase with respect to current are also shown.

WNP *MDR*		**Uh=7 m s^{-1} (fast)**	**Uh=5 m s^{-1} (moderate)**	**Uh=3 m s^{-1} (slow)**
**Category 1 38 ms^{-1} (weak)**	OCE current (°C)	0.551	0.731	1.106
	OCE future (°C)	0.671	0.880	1.318
	∆OCE (°C)	0.120	0.150	0.212
	∆OCE (%)	22	20	19
**Category 2 46 ms^{-1}**	OCE current (°C)	0.774	1.024	1.526
	OCE future (°C)	0.908	1.192	1.771
	∆OCE (°C)	0.134	0.168	0.245
	∆OCE (%)	17	16	16
**Category 3 54 ms^{-1} (moderate)**	OCE current (°C)	0.986	1.291	1.904
	OCE future (°C)	1.136	1.480	2.188
	∆OCE (°C)	0.150	0.189	0.284
	∆OCE (%)	15	15	15
**Category 4 64 ms^{-1}**	OCE current (°C)	1.267	1.634	2.401
	OCE future (°C)	1.434	1.849	2.751
	∆OCE (°C)	0.166	0.215	0.351
	∆OCE (%)	13	13	15
**Category 5 72 ms^{-1} (intense)**	OCE current (°C)	1.506	1.925	2.844
	OCE future (°C)	1.690	2.167	3.264
	∆OCE (°C)	0.184	0.242	0.420
	∆OCE (%)	12	13	15

Supplementary Table 4: Comparison of current (2006-2015) and future (2091-2100) MME OCE over the NA MDR for the 15 TC scenarios. OCE trend (future minus current) and the percentage of OCE increase with respect to current are also shown.

NA *MDR*		**Uh=7 m s^{-1} (fast)**	**Uh=5 m s^{-1} (moderate)**	**Uh=3 m s^{-1} (slow)**
**Category 1 38 m s^{-1} (weak)**	OCE current (°C)	0.363	0.490	0.771
	OCE future (°C)	0.467	0.627	0.968
	∆OCE (°C)	0.104	0.137	0.197
	∆OCE (%)	29	28	26
**Category 2 46 m s^{-1}**	OCE current (°C)	0.530	0.719	1.120
	OCE future (°C)	0.655	0.879	1.344
	∆OCE (°C)	0.125	0.160	0.224
	∆OCE (%)	24	22	20
**Category 3 54 m s^{-1} (moderate)**	OCE current (°C)	0.698	0.939	1.445
	OCE future (°C)	0.841	1.120	1.692
	∆OCE (°C)	0.143	0.181	0.247
	∆OCE (%)	20	19	17
**Category 4 64 m s^{-1}**	OCE current (°C)	0.921	1.226	1.867
	OCE future (°C)	1.087	1.431	2.138
	∆OCE (°C)	0.166	0.205	0.271
	∆OCE (%)	18	17	15
**Category 5 72 m s^{-1} (intense)**	OCE current (°C)	1.118	1.474	2.234
	OCE future (°C)	1.299	1.691	2.522
	∆OCE (°C)	0.181	0.217	0.288
	∆OCE (%)	16	15	13

Supplementary Table 5: OCE trend (future minus current) over the WNP MDR, calculated based on initial CMIP2 ocean profile from Knutson et al. (2001) (Supplementary Fig. 5a). The percentage of the OCE changes (i.e., ΔOCE (%)) is with respect to (wrt) the result in Supplementary Table 2. It can be seen that the OCE increase based on the results using CMIP2 profile from Knutson et al. 2001 is only about 30-40% of this work (based on CMIP5 profiles).

WNP *MDR* (Knutson et al. 2001)		**Uh=7 m s^{-1} (fast)**	**Uh=5 m s^{-1} (moderate)**	**Uh=3 m s^{-1} (slow)**
**Category 1 38 m s^{-1} (weak)**	ΔOCE (°C)	0.047	0.059	0.086
	ΔOCE (%) wrt CMIP5	39	39	41
**Category 2 46 m s^{-1}**	ΔOCE (°C)	0.048	0.061	0.089
	ΔOCE (%) wrt CMIP5	36	36	36
**Category 3 54 m s^{-1} (moderate)**	ΔOCE (°C)	0.051	0.064	0.094
	ΔOCE (%) wrt CMIP5	34	34	33
**Category 4 64 m s^{-1}**	ΔOCE (°C)	0.052	0.067	0.100
	ΔOCE (%) wrt CMIP5	31	31	28
**Category 5 72 m s^{-1} (intense)**	ΔOCE (°C)	0.053	0.067	0.105
	ΔOCE (%) wrt CMIP5	29	28	25

Supplementary Table 6: OCE trend (future minus current) over the NA MDR, calculated based on initial CMIP2 ocean profile from Knutson et al. (2001) (Supplementary Fig. 5b). The percentage of the OCE changes (i.e., ΔOCE (%)) is with respect to (wrt) the result in Supplementary Table 4. It can be seen that the OCE increase based on the results using CMIP2 profile from Knutson et al. 2001 is only about 30-40% of this work (based on CMIP5 profiles).

NA *MDR* (Knutson et al. 2001)		**Uh = 7 m s^{-1} (fast)**	**Uh = 5 m s^{-1} (moderate)**	**Uh = 3 m s^{-1} (slow)**
**Category 1 38 m s^{-1} (weak)**	ΔOCE (°C)	0.044	0.056	0.079
	ΔOCE (%) wrt CMIP5	42	41	40
**Category 2 46 m s^{-1}**	ΔOCE (°C)	0.046	0.058	0.084
	ΔOCE (%) wrt CMIP5	37	36	38
**Category 3 54 m s^{-1} (moderate)**	ΔOCE (°C)	0.048	0.061	0.088
	ΔOCE (%) wrt CMIP5	34	34	36
**Category 4 64 m s^{-1}**	ΔOCE (°C)	0.051	0.065	0.095
	ΔOCE (%) wrt CMIP5	31	32	35
**Category 5 72 m s^{-1} (intense)**	ΔOCE (°C)	0.053	0.067	0.097
	ΔOCE (%) wrt CMIP5	29	31	34

Supplementary Table 7: Comparison of the normalised OCE trend (OCE increase per degree C SST warming) between this work and results using the Knutson et al. 2001 profile, over the WNP MDR. The ratio results are the percentage of the normalised OCE trend from Knutson et al. 2001 profile with respect to this work. It can be seen that the OCE increase from Knutson et al. 2001 profile is about 40-50% of this work.

**WNP MDR**		**Uh = 7 m s^{-1} (fast)**	**Uh = 5 m s^{-1} (moderate)**	**Uh = 3 m s^{-1} (slow)**
**Category 1 38 m s^{-1} (weak)**	Knutson 2001	0.021	0.027	0.039
	This work	0.040	0.050	0.071
	Ratio (%)	53	53	55
**Category 2 46 m s^{-1}**	Knutson 2001	0.022	0.027	0.040
	This work	0.045	0.056	0.082
	Ratio (%)	48	49	49
**Category 3 54 m s^{-1} (moderate)**	Knutson 2001	0.023	0.029	0.042
	This work	0.050	0.063	0.095
	Ratio (%)	46	46	45
**Category 4 64 m s^{-1}**	Knutson 2001	0.023	0.030	0.045
	This work	0.056	0.072	0.117
	Ratio (%)	42	42	38
**Category 5 72 m s^{-1} (intense)**	Knutson 2001	0.024	0.030	0.047
	This work	0.062	0.081	0.140
	Ratio (%)	39	37	34

Supplementary Table 8: Comparison of the normalised OCE trend (OCE increase per degree C SST warming) between this work and results using the Knutson et al. 2001 profile, over the NA MDR. The ratio results are the percentage of the normalised OCE trend from Knutson et al. 2001 profile with respect to this work. It can be seen that the OCE increase from Knutson et al. 2001 profile is about 40-50% of this work.

**NA MDR**		**Uh=7 m s^{-1} (fast)**	**Uh=5 m s^{-1} (moderate)**	**Uh=3 m s^{-1} (slow)**
**Category 1 38 m s^{-1} (weak)**	Knutson 2001	0.020	0.026	0.036
	This work	0.035	0.046	0.066
	Ratio (%)	58	56	55
**Category 2 46 m s^{-1}**	Knutson 2001	0.021	0.027	0.039
	This work	0.042	0.054	0.075
	Ratio (%)	51	50	52
**Category 3 54 m s^{-1} (moderate)**	Knutson 2001	0.022	0.028	0.041
	This work	0.048	0.061	0.083
	Ratio (%)	46	46	49
**Category 4 64 m s^{-1}**	Knutson 2001	0.024	0.030	0.044
	This work	0.056	0.069	0.091
	Ratio (%)	42	44	48
**Category 5 72 m s^{-1} (intense)**	Knutson 2001	0.024	0.031	0.045
	This work	0.061	0.073	0.097
	Ratio (%)	40	43	46

Supplementary Discussion

Our scientific question is: given the CMIP5 projected change in future subsurface ocean environment, what would be the corresponding impact on the TC-induced ocean cooling effect (OCE), and on TC intensification. Because CMIP5 models do not produce realistic TC intensity, there could also be deficiency in CMIP5 TC track and frequency simulations, as compared to observations[2,3], we do not use TC data from the CMIP5 models. We only use the large-scale oceanic and atmospheric environmental fields of the 22 CMIP5 models under the RCP 8.5 projection.

Instead of using the CMIP5 TC data, we test 15 representative TC scenarios based on 5 TC intensity categories (category 1 to 5 in the Saffir-Simpson scale) and 3 translation (travelling) speed combinations (3, 5, and 7 m s^{-1}, i.e., slow, moderate, and fast travelling), so that a wide spectrum of possible TC scenarios can be covered (Supplementary Table 2). These scenarios do not investigate TC track data. For each scenario, the same TC intensity and translation speed is applied to each ocean grid. The 3DPWP ocean mixed layer model is then run individually and independently at each grid to obtain the TC-induced OCE. The only changing environment condition is the initial ocean profile (from the CMIP5 environment) at each grid.

For example, under scenario 8 (Supplementary Table 2), OCE at each grid is calculated using the same category 3 and 5 m s^{-1} TC translation speed. This addresses the question, given such TC forcing, what would be the corresponding OCE at each grid. Because at each grid, the initial ocean profile is updated each year (from 2006 to 2100), we can then obtain the change in OCE as a time series, according to the change in the initial ocean environment, under consistent TC forcing (category 3) and translation speed (5 m s^{-1}).

The grid size used is 2-degree in Lon/Lat. The two study regions are the western North Pacific (WNP) and the North Atlantic (NA). The initial, pre-TC ocean profile at each grid is the boreal TC-season (July-October) averaged profile from the CMIP5 ocean field in each year. As we have 22 CMIP5 ocean fields, the above method is applied to each of the CMIP5 members and at each grid. After obtaining the OCE results for each grid and from each individual member, a multi-model ensemble OCE is obtained based on averaging the OCE results from each member at each grid. Therefore, we are able to obtain not only the multi-model ensemble mean (MME) but also the spread in OCE due the differences in the 22 different ocean fields at each grid.

We have run the 3DPWP model at each grid, for each year (2006 to 2100), over each of

the 22 CMIP5 ocean fields, to calculate the TC-induced ocean cooling effect. This is done for both WNP and NA, and for each of the 15 scenarios. Because of the large member of such calculation and the use of CMIP5 data, we believe this is one of the most comprehensive OCE assessments so far obtained for TC global warming research.

The objective of this research is to examine the change in OCE due to the change in future initial ocean conditions under the CMIP5 RCP8.5 scenario. Therefore, we do not add-on the possible change in future TC attributes under global warming. Certainly, it is possible that future change in TC attributes may further modify the OCE change. However, at present, we do not even have a clean, baseline OCE change due to change in the future ocean environment alone. Therefore, we are hesitant to introduce further TC changes into the analyses, especially given the large uncertainty in future TC activity projection. In short, the objective of this research is to examine the OCE change due to change in the initial future ocean environmental condition, but without co-varying the TC attribute changes. With the ongoing improvement in TC attribute projection, the co-varying aspect in TC attributes change can then be added to this baseline assessment in subsequent research.

The advantage of our idealized approach (i.e. the 15 hypothetical TC scenarios, Supplementary Table 2) versus the use of CMIP5 TC track data is that OCE can be obtained under strong-enough intensity, as well as covering a wide range of TC conditions. Also, the same TC parameters are applied across the 22 different ocean fields, so that the OCE from the 22 different CMIP5 models can be compared under consistent TC conditions. As Carmago et al.[2] points out, TC frequency in CMIP5 models is typically less than observed, and the intensities are much less than observed. Therefore, the approach of using CMIP5-based TC tracks may have issue of under-sampling of OCE, since only a few grids in the domain have TC entries. Our approach calculates OCE on each grid in the domain, thus to maximise sampling.

The disadvantage of our approach is that there is no track information and uniform TC parameters are applied throughout. Therefore for each scenario, the OCE change under global warming is only from change in ocean condition, but without contribution from possible change in TC attributes, i.e. without the co-varying TC attribute change. As discussed in above, given the issue with TC attribute projection in CMIP5, this work aims to obtain first a well-defined baseline, given future ocean condition change only. With the ongoing improvement in TC attribute projection, the co-varying aspect in TC attributes change can be added to this baseline assessment in subsequent research.

As compared to the dynamical-downscaling approach[4-7], our approach has the advantage of efficiency, and can be applied across 22 CMIP5 ocean fields to assess the spread among ensemble members under TC-ocean coupling condition. Because dynamical-downscaling is a much more expensive and computational-consuming approach, it is more difficult to apply to so many ocean fields individually to compare the performance across the 22 ocean fields (Fig. 4 in the main text and Supplementary Figs. 1–3, 9,10,19).

As from above, our potential intensity approach is not to replace dynamical-downscaling approach. Rather, it aims to complement the much more expensive dynamical-downscaling approach, because our approach can be applied to each of the 22 individual CMIP5 environmental fields to assess the model-to-model dependence. From Bender et al. 2010[5] and Knutson et al.[6,7], the ocean field of the dynamical-downscaling approach is mainly based on the MME from *CMIP3* models and the ocean coupling effect spread across the different models is not assessed. Therefore, we examine the OCE condition for both MME as well as the spread in OCE from the 22 CMIP5 individual members, so that uncertainty in OCE due to model-to-model difference in the 22 ocean environmental fields can be obtained. To the best of our knowledge, we do not find other existing literature showing the TC-ocean coupling effect assessed across so many different CMIP5 fields (Fig. 4 in the main text and Supplementary Figs. 1–3,9,10,19).

Please also kindly note that the dynamical-downscaling approach for the Atlantic in Bender et al. [5] and Knutson et al.[7] are based on *CMIP3* MME ocean subsurface temperature gradient fields. In Bender et al.[5], CMIP 3 *oceanic* and *atmospheric* fields were used. In Knutson et al.[6], *CMIP5 atmospheric* field is used but the ocean subsurface temperature gradient field is based on the *CMIP3 MME oceanic* field (based on MME of 18 CMIP3 models). We thus want to conduct a systematic PI approach to use both *ocean* and *atmospheric* fields from a large representation (22) of the latest *CMIP5* models. Please note that in Knutson et al.[7], the across model spread (10 models) are examined for the CMIP3 atmospheric models, but not in the ocean subsurface fields, since the same MME ocean subsurface temperature gradient field is used.

Another issue to note is that we are not sure whether the under-sampling of TC track and frequency issue in CMIP5 may also affect the OCE sampling in the dynamical-downscaling approach[2], since dynamical-downscaling approach is embedded in the CMIP atmospheric environment. It is uncertain to us that whether there will be an issue on the under-sampling of OCE in these approaches. As a result, we do what we can to cover as much sampling in OCE across 22 CMIP5 models and across 15 scenarios (Supplementary Figs. 7, 8, 15–18).

Finally, with regards to the TC tracks, all existing PI approach (e.g. in Vecchi and Soden 2007[8], Fig. S2 in Bender et al. 2010[5]; and Fig. 9 in Knutson et al 2013[7]) do not include the TC tracks and frequency, since PI approach in itself is to assess how ocean and atmospheric thermodynamic environment is allowing a TC to intensify. Therefore, the PI approach in itself does not involve with TC tracks. However, the original PI (i.e. SST_PI) is based on pre-TC, initial SST and does not include the ocean's subsurface contribution or OCE. Therefore, we include the OCE assessments under 15 different TC-ocean coupling scenarios based on OC_PI, so that impact on PI under a wide-spectrum of TC-ocean coupling scenarios can be obtained (Supplementary Figs. 7, 8, 15–18).

Supplementary References

1 Lin, I. I. *et al.* An ocean coupling potential intensity index for tropical cyclones. *Geophys. Res. Lett.* **40**, 1878–1882 (2013).

2 Camargo, S. J. Global and Regional Aspects of Tropical Cyclone Activity in the CMIP5 Models. *J. Climate* **26**, 9880–9902 (2013).

3 Tory, K. J., Chand, S. S., McBride, J. L., Ye, H. & Dare, R. A. Projected changes in late-twenty-first-century tropical cyclone frequency in 13 coupled climate models from phase 5 of the coupled model intercomparison project. *J. Climate* **26**, 9946–9959 (2013).

4 Knutson, T. R., Tuleya, R. E., Shen, W. & Ginis, I. Impact of CO_2-induced warming on hurricane intensities as simulated in a hurricane model with ocean coupling. *J. Climate* **14**, 2458–2468 (2001).

5 Bender, M. A. *et al.* Modeled impact of anthropogenic warming on the frequency of intense Atlantic hurricanes. *Science* **327**, 454–458 (2010).

6 Knutson, T. R. *et al.* Tropical cyclones and climate change. *Nature Geoscience* **3**, 157–163 (2010).

7 Knutson, T. R. *et al.* Dynamical downscaling projections of twenty-first-century Atlantic hurricane activity: CMIP3 and CMIP5 model-based scenarios. *J. Climate* **26**, 6591–6617 (2013).

8 Vecchi, G. A. & Soden, B. J. Effect of remote sea surface temperature change on tropical cyclone potential intensity. *Nature* **450**, 1066–1070 (2007).
